# Degradation of Redox-Sensitive Proteins including Peroxiredoxins and DJ-1 is Promoted by Oxidation-induced Conformational Changes and Ubiquitination

**DOI:** 10.1038/srep34432

**Published:** 2016-10-05

**Authors:** In-Kang Song, Jae-Jin Lee, Jin-Hwan Cho, Jihye Jeong, Dong-Hae Shin, Kong-Joo Lee

**Affiliations:** 1 Graduate School of Pharmaceutical Sciences and College of Pharmacy, Ewha Womans University, Seoul 03760, Republic of Korea

## Abstract

Reactive oxygen species (ROS) are key molecules regulating various cellular processes. However, what the cellular targets of ROS are and how their functions are regulated is unclear. This study explored the cellular proteomic changes in response to oxidative stress using H_2_O_2_ in dose- and recovery time-dependent ways. We found discernible changes in 76 proteins appearing as 103 spots on 2D-PAGE. Of these, Prxs, DJ-1, UCH-L3 and Rla0 are readily oxidized in response to mild H_2_O_2_ stress, and then degraded and active proteins are newly synthesized during recovery. In studies designed to understand the degradation process, multiple cellular modifications of redox-sensitive proteins were identified by peptide sequencing with nanoUPLC-ESI-q-TOF tandem mass spectrometry and the oxidative structural changes of Prx2 explored employing hydrogen/deuterium exchange-mass spectrometry (HDX-MS). We found that hydrogen/deuterium exchange rate increased in C-terminal region of oxidized Prx2, suggesting the exposure of this region to solvent under oxidation. We also found that Lys191 residue in this exposed C-terminal region of oxidized Prx2 is polyubiquitinated and the ubiquitinated Prx2 is readily degraded in proteasome and autophagy. These findings suggest that oxidation-induced ubiquitination and degradation can be a quality control mechanism of oxidized redox-sensitive proteins including Prxs and DJ-1.

Reactive oxygen species (ROS) including superoxide anion (O_2_^−^), hydrogen peroxide (H_2_O_2_), and hydroxyl radical (OH∙), act on lipids, proteins, DNA and their targets and cause oxidative modifications^1^. Recent studies demonstrated that H_2_O_2_ is a key signaling molecule in redox signaling that promotes cellular processes such as proliferation, differentiation, migration and metastasis, angiogenesis, inflammation and cell death^2^,^3^,^4^,^5^. While low concentrations of H_2_O_2_ stimulate cell proliferation and differentiation^6^, high concentrations can injure cells by oxidizing various cellular components. Disturbance of homeostasis by H_2_O_2_ has been associated with various diseases such as carcinogenesis^7^, neurodegeneration^8^,^9^, atherosclerosis^10^, diabetes^11^, and aging^12^.

Cells maintain homeostasis by tight control of intracellular concentration of H_2_O_2_^13^, by generation of H_2_O_2_ via NADPH oxidases (NOXs), by promoting H_2_O_2_ signaling through direct oxidation of target proteins, and by scavenging H_2_O_2_ via antioxidant enzymes such as peroxiredoxins (Prxs)^14^. However, the molecular mechanisms underlying these H_2_O_2_ actions and their dependence on the degree of oxidative stress are not well understood.

Prxs are Cys-based peroxidases, highly abundant in and conserved in bacteria to humans. They play crucial roles in protecting cells from oxidative stress, and maintain genome stability and longevity^15^,^16^. Prxs have been classified into three types: 1) Prx1–4, known as 2-Cys Prxs, forming homodimer with two conserved cysteine residues; 2) Prx5, known as an atypical 2-Cys Prx, forming intramolecular disulfide bonds with its two Cys residues; and 3) Prx6, 1-Cys Prx containing 1 active Cys residue^17^. Typical 2-Cys Prx has a catalytic cycle with two conserved Cys residues; one is an active site, peroxidative Cys (C_P_) and the other, a resolving Cys (C_R_). Peroxidatic Cys is sensitive to oxidative stress, and is oxidized by H_2_O_2_ to cysteine sulfenic acid (Cys-SOH), which is linked to resolving Cys, forming disulfide bond^18^. The catalytic cycle is completed by reducing the disulfide bond of Prxs by thioredoxin (Trx) in NADPH-dependent thioredoxin reductase-thioredoxin system^19^. Peroxidative Cys of 2-Cys Prxs, which is oxidized to sulfenic acid (C_P_-SOH), can be further oxidized by a second H_2_O_2_ molecule to cysteine sulfinic acid (C_P_-SO_2_H)^20^. The Prxs, hyperoxidized to sulfinic acid, not sulfonic acid, can be reduced to active form by sulfiredoxin^21^. 1-Cys Prx6 is known to be oxidized to various oxidation states at Cys47, including sulfenic, sulfinic, sulfonic acids; Cys to dehydroalanine (Dha), to Ser, to thiosulfonate (C_P_-SO_2_SH) and to many unknown states including +134, 150 and +284 Da changes^22^. DJ-1, presumed to be an antioxidant protein, is believed to be related to Parkinson’s disease (PD)^23^, because DJ-1 mutants cause the neuronal diseases^24^. However, the role of DJ-1 under oxidative stress is not well understood.

Delineation of proteomic changes occurring in cellular proteins in response to H_2_O_2_ stress can lead to understanding of the mechanism underlying H_2_O_2_-mediated signaling pathway. To understand the molecular action of H_2_O_2_ in target proteins, it is necessary to identify the oxidative modifications in redox-sensitive Cys residues. Recently, employing the SEMSA proteomic strategy for identifying low abundant post-translational modifications (PTM) using nanoUPLC-ESI-q-TOF tandem mass spectrometry^25^ in combination with PTM search algorithm MOD^i^ 26, the diverse and novel oxidative modifications in redox-sensitive proteins have been delineated. It is well known that redox-sensitive Cys residues are readily oxidized to disulfide, sulfenic acid (SOH), sulfinic acid (SO_2_H) and sulfonic acid (SO_3_H). Furthermore, novel oxidative modifications of redox-sensitive Cys residue including thiosulfonate crosslinking (Cys-SO_2_-S-Cys), Cys to thiosulfonic acid (Cys-SO_2_-SH, Δm = +64 Da), serine (Ser, Δm = −16 Da), and dehydroalanine (Dha, Δm = −34 Da) have been identified^27^. In order to find the ROS target proteins having redox-sensitive Cys, a novel chemical labeling reagent, methyl-3-nitro-4-(piperidin-1-ylsulfonyl) benzoate (NPSB-1) that selectively and specifically reacts with sulfhydryl of redox-sensitive Cys residues, has been designed and synthesized^28^. Employing biotinylated NPSB-1 (NPSB-B), the H_2_O_2_ sensitivities of Cys residues were measured and 226 H_2_O_2_-sensitive proteins identified. Of these, some proteins having reactive Cys residues are easily oxidized by H_2_O_2_ as expected, but some proteins not having any reactive Cys residues are converted to reactive ones by mild oxidations. An explanation for this is that mild oxidation induces the structural changes which convert the non-reactive Cys residue to reactive one. As an example, it was demonstrated in Nm23-H1, a metastasis suppressor protein, that formation of disulfide bond between Cys4 and Cys145 induces structural changes, which cause Cys109 residue to be readily oxidized to various oxidation states in stepwise oxidations^29^,^30^. These oxidative modifications of redox-sensitive proteins are presumed to play significant but poorly understood roles in cellular functions and regulations of proteins. Further studies on the actions of ROS at molecular level, so called ‘ROSics’, are needed for identifying redox-sensitive Cys residues, their oxidative modifications and structural changes caused by oxidation, and understanding how biological activities are regulated by oxidation^31^.

In this study, we separated ROS-treated cellular proteins employing 2D gel electrophoresis, identified ROS target proteins and their various modifications using nanoUPLC-ESI-q-TOF tandem MS, and characterized many redox-sensitive proteins as well as their diverse modifications responding to H_2_O_2_. We found that redox-sensitive proteins including Prxs, DJ-1, UCH-L3 and Rla0 regulate protein stability through oxidative modifications, and that oxidized proteins are readily degraded by proteasome and autophagy. In order to explain how oxidation induces protein degradation, oxidation-induced structural changes were identified employing hydrogen/deuterium exchange-mass spectrometry (HDX-MS) in redox-sensitive Prx2 protein. HDX-MS showed that oxidation induced conformational changes of Prx2 exposed its C-terminal region to the solvent, causing Lys191 residue in the C-terminal region to be ubiquitinated, and the ubiquitinated Prx2 to be readily degraded in proteasome and autophagy. This study thus suggests that oxidative modifications and oxidative stress-induced structural changes of some redox-sensitive proteins play important roles in homeostasis of proteins.

## Results

### Identification of proteins differentially appearing in MDA-MB-231 cells following H_2_O_2_ treatment

**Figure 1:**
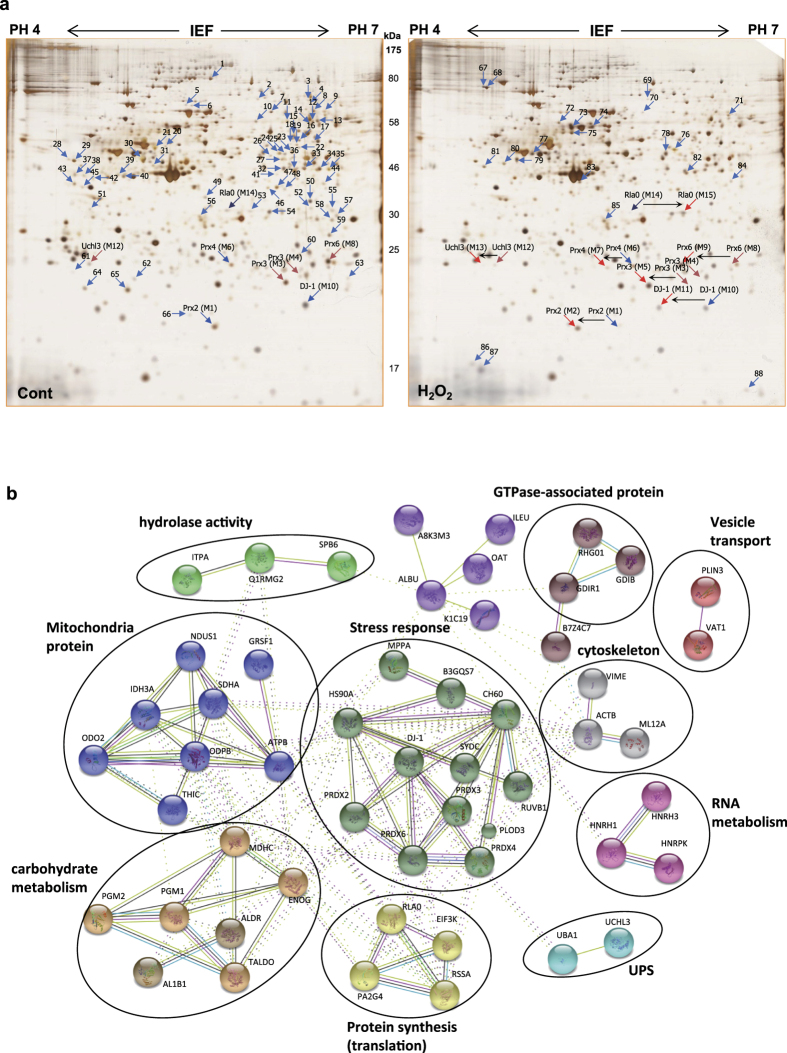
Differential protein expression in MDA-MB-231 cells treated with H_2_O_2_. (**a**) 2D-PAGE separation and silver staining of MDA-MB-231 cells treated by control (left) and 0.5 mM H_2_O_2_ for 30 min (right). (**b**) Proteins altered by H_2_O_2_ treatment (Supplementary Table 1) were analyzed using protein interaction data retrieved from STRING. Lines indicate protein-protein interactions with confidence coefficient higher than 0.7.

**Figure 2:**
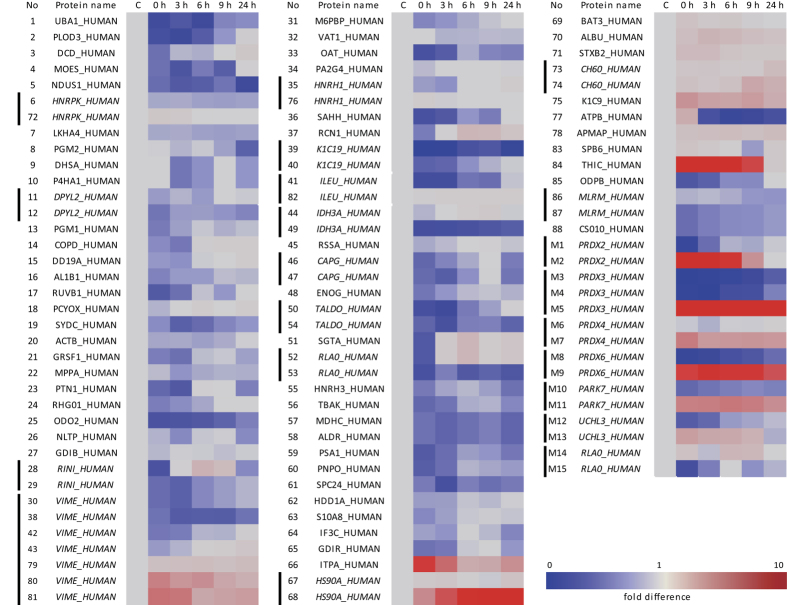
Heat map of kinetics of differentially expressed proteins during recovery in control media after 0.5 mM H_2_O_2_ treatment. Vertical line in left side indicates the same protein appearing in several spots on 2D-PAGE.

**Figure 3:**
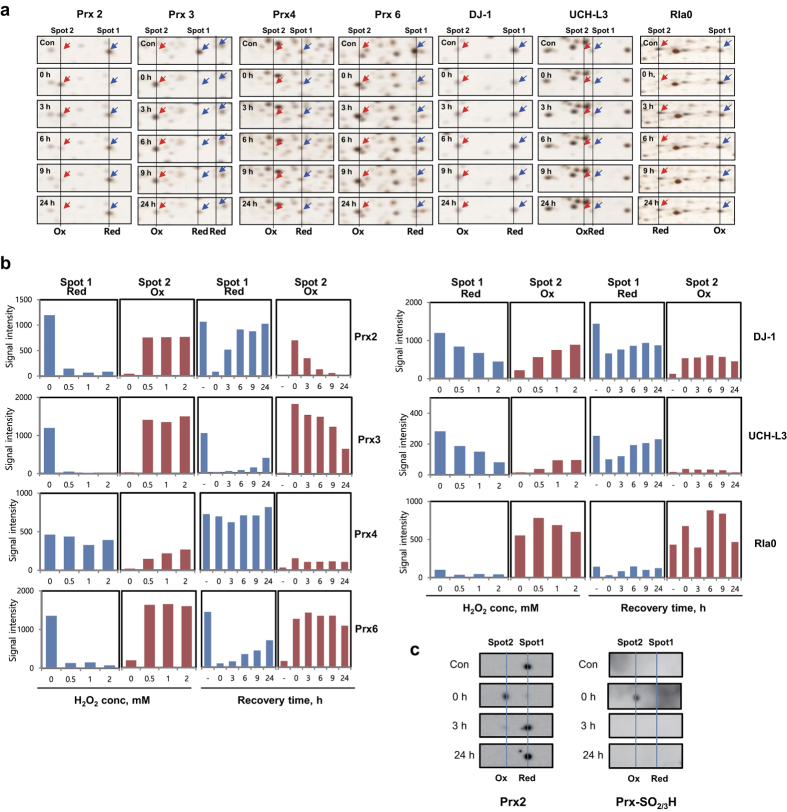
Changes in modified redox-sensitive proteins during recovery after H_2_O_2_ treatment. (**a**) Representative sliver-stained 2D-gel images on a non-linear pH gradient (4–7) of each redox-sensitive proteins in MDA-MB-231 cells during recovery after treatment (control or 0.5 mM H_2_O_2_ in HBSS for 30 min). (**b**) Each protein spot at reduced and oxidized status was quantified and data from triplicate experiments are presented. (**c**) Western analysis of Prx2 in (**a**) sample during recovery time after 0.5 mM H_2_O_2_ treatment using anti-Prx2 antibody (left panel) and anti-Prx-SO_2/3_H antibody (right panel).

We examined the protein profiles of MDA-MB-231 invasive breast cancer cell line, after treatment with various concentrations of H_2_O_2_, and during recovery after H_2_O_2_ treatment, by proteomic analysis after protein separations on 2D-PAGE. We identified the proteins differentially appearing in 2D-PAGE, employing peptide sequencing with nanoUPLC-ESI-q-TOF tandem MS (MS/MS). This approach demonstrated that 103 protein spots from 2D-PAGE, were altered in response to 0.5 mM H_2_O_2_ treatment for 30 min, which we found to be the optimal non-toxic conditions for studying H_2_O_2_ effect in this cell line (Fig. 1a). Each of these spots was identified by MS/MS and presented in Supplementary Table S1, along with accession numbers, mascot scores, queries matched peptides, theoretical molecular weights/pIs, and their fold changes compared to control of the identified proteins. Differentially appearing proteins were classified by STRING analysis based on interacting proteins (Fig. 1b). Intensity fold changes of each spot during recovery after H_2_O_2_ treatment were expressed as heat map (Fig. 2). This study showed that the functions of protein targets of H_2_O_2_ could be divided into two groups: one group of proteins closely associated with each other based on protein-protein interactions, is involved in stress response, mitochondria, carbohydrate metabolism, protein synthesis, UPS, RNA metabolism and cytoskeleton. The second group of non-associated proteins is GTPase-related proteins or act in vesicle transport and hydrolase activity. Among the 76 proteins identified, seven redox-sensitive proteins (Prx2, 3, 4, 6, DJ-1, UCH-L3 and Rla0) were significantly changed in pIs in response to H_2_O_2_ treatment (Fig. 1a, Supplementary Figure S1). Protein spots of Prx2, 3, 4, 6, DJ-1 and UCH-L3, were shifted to left (to more acidic region) by oxidative stress, while protein spots of Rla0, 60S acidic ribosomal protein P0, which moved to right (more basic region). As shown in Fig. 3b, spots of oxidized Prx4 and DJ-1 increased in a H_2_O_2_ dose dependent manner, while those of Prx2, 3, 6 and were saturated at low concentration 0.5 mM H_2_O_2_ treatment. Based on this study, we selected 0.5 mM H_2_O_2_ treatment for kinetic studies.

We determined recovery kinetics after oxidative stress, based on the spot shift. For this, MDA-MB-231 cells were treated with 0.5 mM H_2_O_2_ in HBSS for 1 h at 37 °C, washed and then recovered in fresh media for indicated times. Representative protein profiles are compared in Fig. 1a. Protein spots of Prx2, 3, 4, 6, DJ-1, UCH-L3 and Rla0 on silver stained 2D-PAGE in each recovery time point are shown in Fig. 3a and triplicate samples semi-quantitated by image analysis using software Progenesis SamSpots V 4.5 are shown in Fig. 3b. Recovery rates of redox-sensitive proteins varied: oxidized spots of Prx2, 3 and 6 rapidly disappeared and reduced spot Prx2 reappeared in 3 h, while recovery rates of Prx3, Prx6, UCH-L3 and Rla0 were relatively slower than Prx2. Prx4 existing in ER in oxidizing environment was not changed by oxidative stress. Prx1, which is a basic protein (pI = 8.27), was not detected on 2D-PAGE (pI range, 4–7). These pI shifts of redox-sensitive proteins in response to oxidative stress and during recovery time, were confirmed using 2D western blot analysis with anti-Prx2 antibody and with anti Prx-SO_2/3_H antibody (Fig. 3c). These results indicate that Prx2, Prx3, Prx6, DJ-1 and UCH-L3 were readily oxidized by oxidative stress, and were quickly restored to reduced form inside cells.

### Post-translational modifications of redox-sensitive proteins

**Figure 4:**
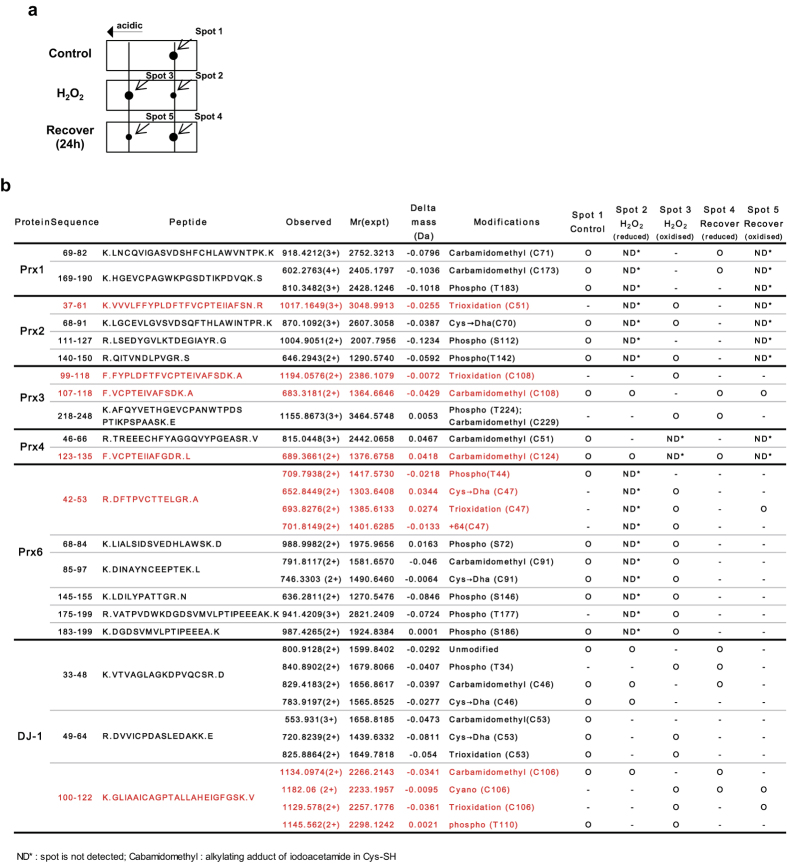
Post-translational modifications of redox-sensitive proteins in 2D-PAGE spots. (**a**) 2D-PAGE diagram of spot changes of redox-sensitive proteins after 0.5 mM H_2_O_2_ treatment for 30 min. Spot 1 is an original control protein. Spot 2 is a control spot and spot 3 an oxidized one shifted to acidic side, after H_2_O_2_ treatment. Spot 4 is an original protein and spot 5 is an oxidized one in 24 h recovery time after H_2_O_2_ treatment. (**b**) Identification of protein modifications in each spot of redox-sensitive proteins in (**a**) by peptide sequencing with MS/MS.

In order to investigate protein modifications resulting in response to oxidative stress, we comprehensively examined control and oxidized spots of redox-sensitive proteins by peptide sequencing with nanoUPLC-ESI-q-TOF MS/MS, employing SEMSA, which is sensitive technique for identifying low abundant modifications^25^, and novel modifications were identified for using MOD^i^ and MODmap searching algorithms^26^,^32^,^33^. Diversely modified populations, which were repeatedly detected more than 3 times, were identified in various peptides at specific sites (Fig. 4 and Supplementary Figure S2). We found that the peptides in spot 3 (Fig. 4a) obtained after H_2_O_2_ treatment, shifted to left, indicating trioxidation, phosphorylation and other newly identified oxidative modifications of Cys to dehydroalanine (Dha) (Δm = −34 Da) and thiosulfonic acid (Δm = +64 Da)^27^, in contrast to peptides in control spot 1. Specifically, oxidized spot 3 of Prx2 showed trioxidation at active site Cys51 of peptide ^37^VVVLFFYPLDFTFVCPTEIIAFSN^61^ (m/z = 3051.4947 Da, z = 1) and Prx3 spot 3 showed trioxidation at Cys108 of peptide ^99^FYPLDFTFVCPTEIVAFSDK^118^ (m/z = 2388.1152 Da, z = 1) containing active site and phosphorylation at Tyr224. Prx6 spot 3 showed various modifications including conversion of active site Cys47 to Dha, trioxidation and thiosulfonic acid in peptide ^42^DFTPVCTTELGR^53^ (m/z = 1339.6898 Da, z = 1), conversion of regulatory Cys91 to Dha in peptide ^85^DINAYNCEEPTE^96^ (m/z = 1492.6606 Da, z = 1) and phosphorylations at various sites including Ser146, Tyr177 and Ser186. DJ-1 spot 3 showed various modifications including oxidation at active site Cys106 to thiocyanide (-SCN, Δm = 25 Da) and trioxidation, and phosphorylation at Tyr110 in peptide ^100^GLIAAICAGPTALLAHEIGFGSK^122^ (m/z = 2163.156 Da, z = 1), and conversion of regulatory Cys53 to Dha in peptide ^49^DVVICPDASLEDAKK^64^ (m/z = 1441.6478 Da, z = 1). These findings suggest that oxidation-induced acidic shifts on 2D-PAGE originated from modifications such as Cys conversion to various oxidative states and phosphorylations. Further studies are needed to elucidate the functional changes from these modifications.

### Redox- sensitive proteins are readily degraded by oxidation

**Figure 5:**
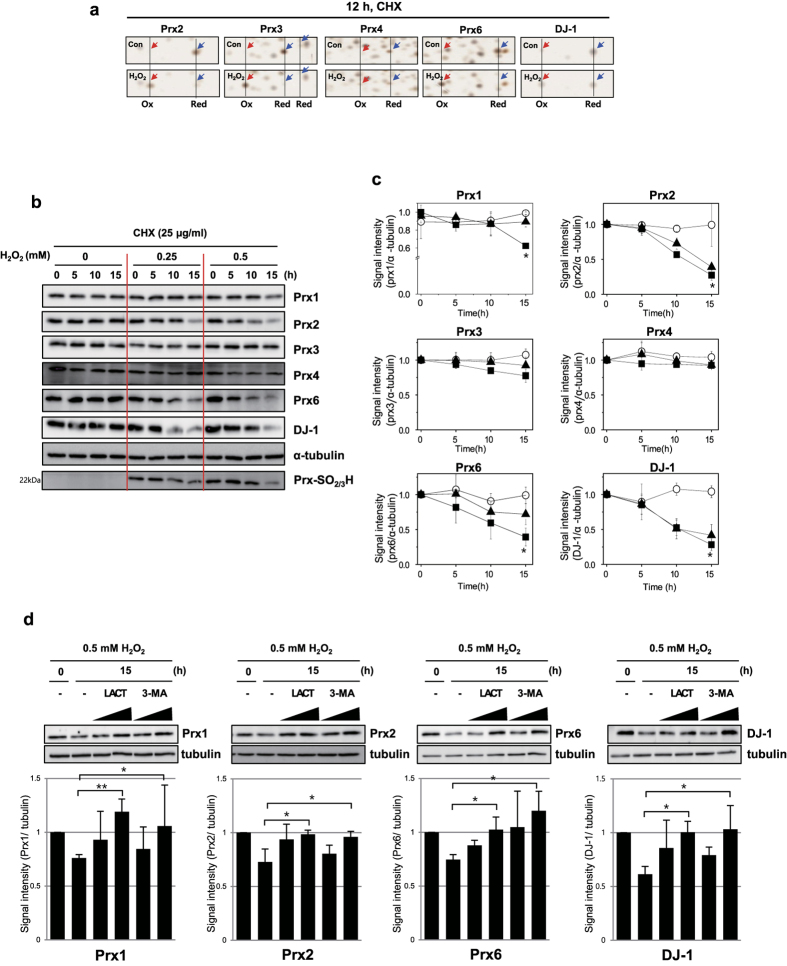
Degradation of redox-sensitive proteins under oxidative stress. (**a**) 2D-gel images of Prxs and DJ-1 in MDA-MB-231 cells during recovery with CHX after H_2_O_2_ treatment. MDA-MB-231 cells treated with 0.5 mM H_2_O_2_ in HBSS for 1 h, were washed, and incubated for 12 h in EMEM supplemented with 10% FBS, with CHX (25 μg/mL). (**b**,**c**) Degradation kinetics of Prxs and DJ-1 were confirmed by western analysis. MDA-MB-231 cells treated with various concentrations of H_2_O_2_ (0 mM (○), 0.25 mM (▲), 0.5 mM (■)), were incubated in EMEM supplemented with 10% FBS, with CHX (25 μg/mL) for indicated time. Cell lysates were separated on SDS-PAGE and detected with western analysis using various antibodies (**b**). Quantified data of (**b**) were presented as graph (**c**). *p < 0.05; **p < 0.01 versus control. (**d**) Degradations of oxidized Prx1, 2, 6 and DJ-1 were blocked by proteasome inhibitor lactacystin and autophagy inhibitor 3-MA. MDA-MB-231 cells treated with 0.5 mM H_2_O_2_ in HBSS for 1 h were incubated for 15 h in EMEM supplemented with 10% FBS, CHX (25 μg/mL) and lactacystin (1 and 5 μm), an irreversible proteasome inhibitor, and 3-MA (1 and 5 mM), an autophagy inhibitor.

We determined whether the reduced spot generated during recovery after oxidative stress originated from reduction of oxidized protein or from a newly synthesized protein by examining the effect of cycloheximide (CHX), an inhibitor of protein synthesis, on its generation. MDA-MB-231 cells were treated with 0.5 mM H_2_O_2_ for 1 h and then recovered with fresh media containing 25 μg/mL CHX for various times. The proteins in the cell lysates were then separated on 2D-PAGE and detected with silver staining. As shown in Fig. 5a, oxidized spots disappeared, but reduced spots did not reappear during recovery with CHX. These results suggest that oxidized forms of redox-sensitive proteins are readily degraded and newly synthesized proteins appeared in reduced forms, indicating another pathway for oxidized Prxs, in addition to Prx1 oxidation to sulfinic acid and then recovery to reduced form by sulfiredoxin^34^.

We confirmed the degradation of redox-sensitive proteins under oxidative condition, with western analysis on 1D-PAGE using specific antibodies. MDA-MB-231 cells were incubated with various concentrations of H_2_O_2_ for 1 h and recovered in fresh media containing 25 μg/mL CHX for indicated times. Redox-sensitive proteins treated with H_2_O_2_ were readily degraded at different rates, depending on the protein species (Fig. 5b,c). Oxidized Prx1, Prx2, Prx6 and DJ-1 were readily degraded, while oxidized Prx3 was degraded slower. However, Prx4 located in ER, a oxidizing environment, was neither oxidized by exogenous H_2_O_2_ (Fig. 3a), nor degraded during recovery (Fig. 5b,c). Among 2-Cys Prxs, Prx3 is more resistant to oxidative stress than Prx1 and Prx2. Prx1 oxidized to disulfide and sulfenic acid is known to be reduced by thioredoxin and sulfiredoxin respectively^35^. Prxs and DJ-1 hyperoxidized to sulfonic acid (Cys-SO_3_H) were detected inside cells, but their regulation mechanisms are unknown. These findings suggest that hyperoxidized proteins are readily degraded inside cells.

### Redox-sensitive proteins are degraded via proteasome and autophagy under oxidative conditions

A recent study demonstrated that nitrosative stress induces Prx1 ubiquitination via E3-ligase E6AP activation^36^. In order to understand how oxidized Prxs are degraded inside cells, we examined the degradation pathways employing inhibitors of proteasome and autophagy. MDA-MB-231 cells were treated with 0.5 mM H_2_O_2_ for 1 h, and recovered for 15 h to obtain the maximum degradation in fresh media containing CHX (25 μg/mL), and various concentrations of lactacystin, an irreversible proteasome inhibitor, or 3-methyladenine (3-MA), an autophagy inhibitor. As shown in Fig. 5d, degradations of oxidized Prx1, Prx2, Prx6 and DJ-1 were significantly inhibited by both lactacystin and 3-MA, while the degrees of inhibition varied depending on the proteins. These results suggest that these oxidized redox-sensitive proteins are cleared by both proteasome and autophagy.

### Structural changes in oxidized Prx2 identified by hydrogen/deuterium exchange-mass spectrometry (HDX-MS)

**Figure 6:**
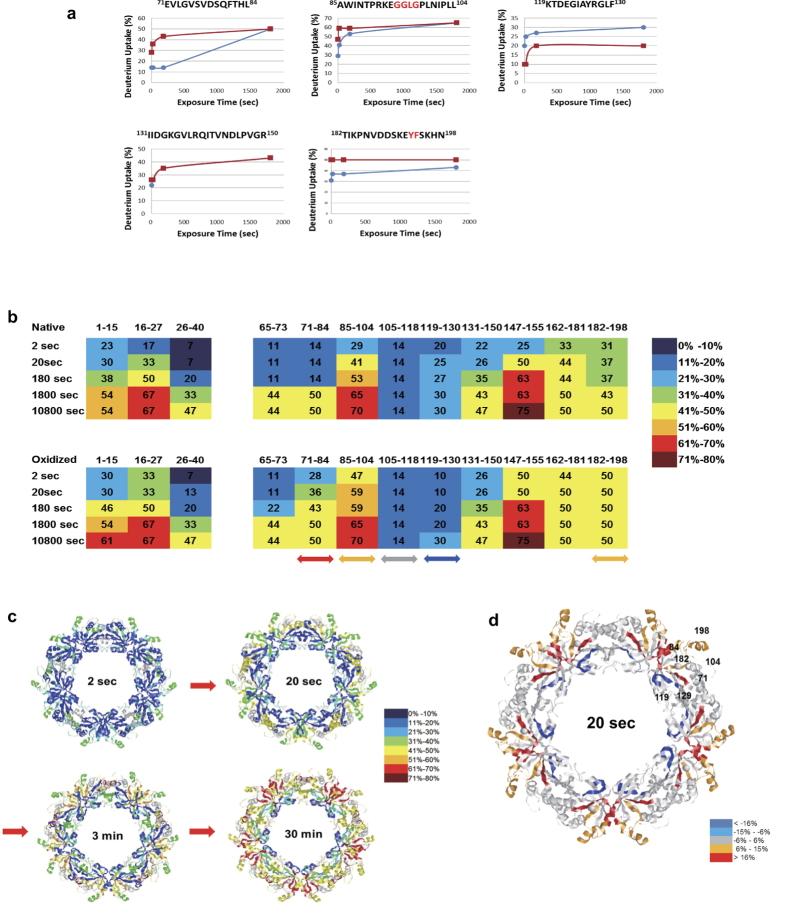
Identification of structural changes in Prx2 under the oxidative stress employing HDX-MS. Recombinant Prx2 treated with and without H_2_O_2_ for 1 h, was incubated with D_2_O exchange buffer at 25 °C for various times upto 3 h and analyzed using nanoAcquity™/ESI/MS. (**a**) Time course of HDX incorporation for representative peptides that showed differences in HDX. (**b**) Deuterium exchange rate (%) of native and oxidized Prx2. Significantly changed representative peptides were marked by colored arrows and gray arrow means no change peptide as control. The corresponding deuterium exchange levels for each peptide in percent are given on the right. (**c**) Changes in HDX-MS of native Prx2 as compared with the 0 s incubation at various incubation times. (**d**) Changes in HDX-MS of oxidized Prx2 compared to the native Prx2 at 20 sec in decamer form.

Because Prx2 was readily oxidized and degraded in response to oxidative stress, we investigated how oxidized redox-sensitive proteins are degraded by examining the conformational changes of Prx2 under oxidative condition, employing hydrogen/deuterium exchange-mass spectrometry (HDX-MS). Recombinant control and oxidized Prx2s were incubated in D_2_O for various times, and hydrogen/deuterium exchange (HDX) ratio was monitored by mass spectrometry in which residues exposed to protein surface more readily exchange deuterium allowing their mass increases to be detected, as described previously^37^. H/D exchange rates of control and oxidized Prx2 were compared in each peptic digested peptide (Fig. 6a and Supplementary Figure S5). Combined stitching the H/D exchange ratios of each peptic peptide showed the diagram of whole protein shown in Fig. 6b. Peptide MS coverage was 88%. More deuterium exchanges in oxidized Prx2 occurred in peptides containing GGLG motif (^85^AWINTPRKEGGLGPLNIPLL^104^) and YF motif (^182^TIKPNVDDSKEYFSKHN^198^), while less deuterium exchange were observed in peptide 119–130. These changed regions were marked in the Prx2 decamer structure (PDB entry 1QMV) in Fig. 6d and compared to control Prx2 in time dependent manner (Fig. 6c). These results demonstrate that dimer-dimer surface regions of Prx2 are significantly changed by oxidation. C-terminus known as a flexible region, and GGLG regions identified as conformational changes during catalysis^38^ are exposed to the protein surface by oxidative stress, while peptide (119–130 aa) having helix-turn-helix region was more shielded in oxidized condition. These results indicate that tertiary and quaternary conformational changes occur in oxidized Prx2.

### Exposure of C-terminal region to solvent by oxidative stress induces polyubiquitination and degradation

**Figure 7:**
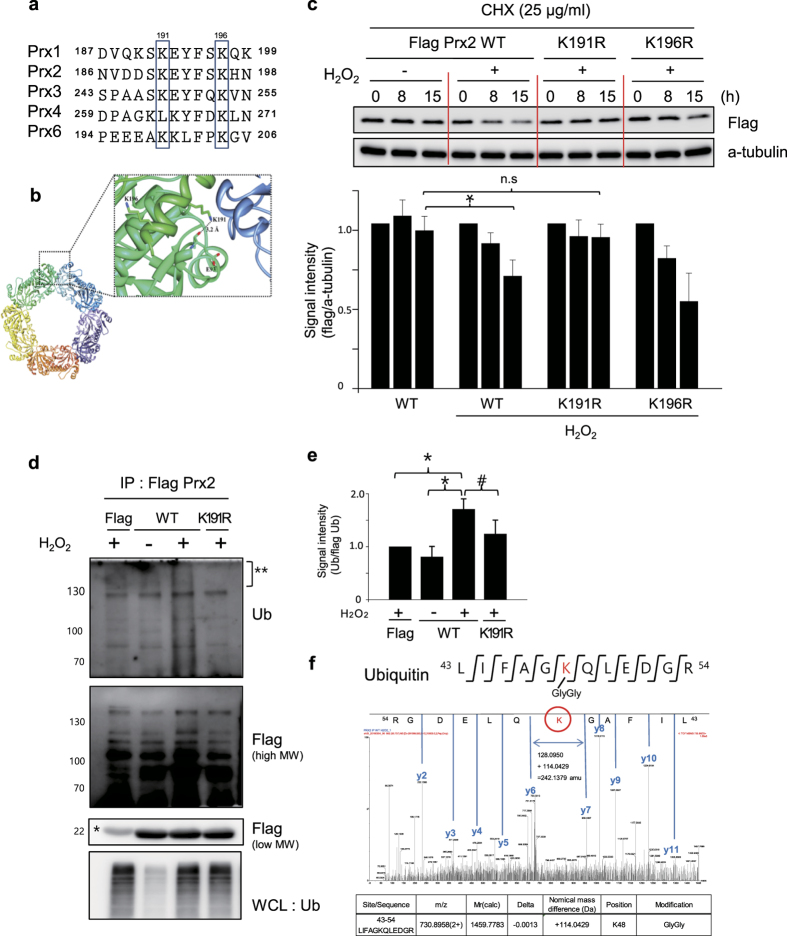
Regulation of stability of oxidized Prx2 by ubiquitination. (**a**) Alignment of amino acid sequences in C-terminal region of Prxs. Lys191 residue of Prx2 is conserved in Prx family. (**b**) The locations of K191 and K196 on reduced Prx2. The five dimers consisting of the decamer is colored differently. K191 and K196 residues located on the C-terminal helix are represented with a stick-model and the hydrogen-bond between K191 and E93 is drawn with a dotted-line. Under oxidative condition, the C-terminal helix of Prx2 proceeds a helix-to-loop transition by a huge quaternary structural change. Therefore, K191 is fully exposed on the surface and freely accessible by solvents. (**c**) Oxidized Prx2 was degraded by polyubiquitination of K191 residues. Hela cells transfected with Flag Prx2 wild type and K191R/K196R mutants, were treated with 0.5 mM H_2_O_2_ in HBSS for 1 h, then incubated in EMEM with 10% FBS and CHX (25 μg/mL) for indicated times. Protein degradations were measured by western analysis (upper panel), and quantified in triplicate experiments (lower panel). *p < 0.05 versus control; n.s: no significant. (**d**) Ubiquitination of wild Prx2 and K191 mutant. Hela cells transfected with Flag, Flag-Prx2, Flag-Prx2 K191R mutant and HA-Ub were treated with H_2_O_2_ for 1 h and recovered for 12 h in media containing CHX, 2.5 μM lactacystin and 2.5 mM 3-MA. Cell lysates were immunoprecipitated with anti-Flag antibody, and immune-complex were separated and detected with western analysis using anti-Ub (upper panel) and anti-Flag antibody (middle panel for high molecular weight Prx2, and lower panel for low molecular weight Prx2). *Non-specific band from IgG antibody. (**e**) Quantitative analysis of polyubiquitinated proteins in each high molecular band in upper panel in Fig. 7d, *p < 0.05 versus control; ^#^p < 0.1 versus control. (**f**) Lys-linkage of polyubiquitinated proteins were identified by MS/MS. Prx2 wild type high molecular band on 1D-PAGE in Fig. 7d (**) was analyzed with nanoUPLC-ESI-q-TOF MS/MS employing SEMSA and MOD^i^. K48-GlyGly (+114.0429 Da) modification was observed in MS/MS spectra.

Since oxidized Prx2 is readily degraded by proteasome and autophagy, we examined the possible ubiquitination sites in the exposed surface region of oxidized Prx2. Using UbPred^39^, we predicted that two lysine residues, K191 and K196, of the oxidized Prx2 peptide sequence were the sites ubiquitinated. These sites are conserved in isoforms of Prx (Fig. 7a). We further analyzed the 3D structures of reduced (decamer) and oxidized (dimer) forms of human Prx2. Since a 3D structure of oxidized Prx2 has not been described, we used MODELLER^40^ to build and compare them. This clearly showed that K196 is exposed on the protein surface in both cases. In contrast, the solvent accessibility to K191 in the reduced form of Prx2 was found to be limited. Under reduced condition, K191 in one subunit of the dimer is hydrogen-bonded to the backbone oxygen of E93 of the other subunit, and is located at the dimer-dimer interface of the decamer (Fig. 7b). Therefore, a substantial quaternary structural change from decamer to dimer, depending on the redox state of Prx2, influences surrounding environment of K191 more markedly than that of K196. C-terminal peptide containing YF motif is discernibly exposed to surface region (Fig. 6b). We therefore examined whether the regulation of oxidized protein degradation by the control and oxidized Prx2, is different.

In order to investigate whether these sites are ubiquitinated, we generated K191R and K196R mutants and examined their degradation rates. Hela cells transfected with wild Flag-Prx2, K191R and K196R mutants were exposed to 0.5 mM H_2_O_2_ for 1 h and then incubated in fresh media containing 25 μg/mL CHX. As shown in Fig. 7c, K191R mutant was stable, and was not degraded under oxidative stress in contrast to the WT and K196R mutants.

In order to investigate whether K191 is involved in the ubiquitination of oxidized Prx2, we examined whether Prx2 and its mutants were ubiquitinated by oxidative stress in Hela cells. Hela cells co-transfected with HA-Ub and Flag-Prx2 wild type, or K191R mutant, were immunoprecipitated with anti-Flag antibody and the immune-complexes were analyzed by western blotting using anti-Ub and anti-Flag antibodies. As shown in Fig. 7d,e, ubiquitinated Prx2, not Prx2 K191R mutant, was detected in cells transfected with Prx2 wild type, after oxidative stress. And these Prx2 ubiquitinations (Fig. 7d**) were identified as K48-linkage in MS/MS spectrum (Fig. 7f). These results demonstrate that the C-terminus of Prx2 is exposed to protein surface after oxidative stress and that the unmasked K191 residue in C-terminus is readily ubiquitinated by E3 ligase and degraded in proteasome and autophagy. This ubiquitination dependent Prx degradation was again confirmed in Prx1, another 2-Cys Prx, as shown in Supplementary Figure S6.

## Discussion

**Figure 8:**
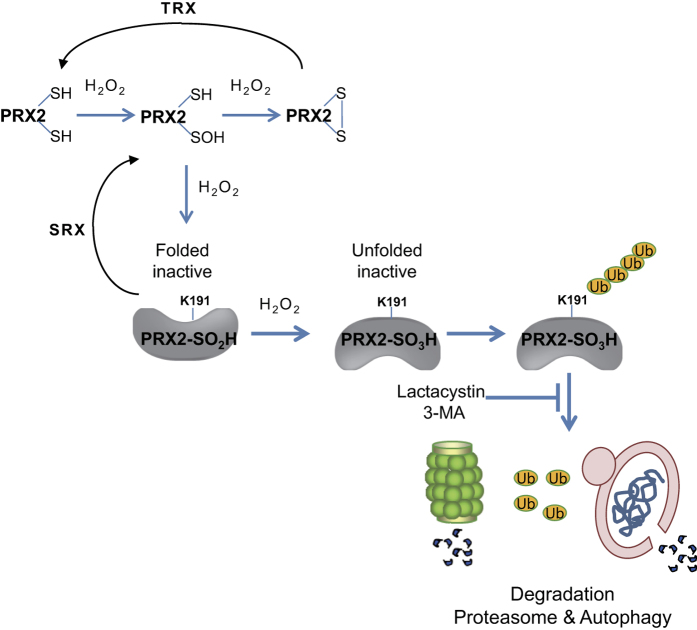
A proposed mechanism for Prx2 hyperoxidation and degradation by proteasome and autophagy.

In the present study, we identified the redox-sensitive proteins in cells exposed to oxidative stress using proteomic tools and found that 76 proteins/103 spots were changed in response to H_2_O_2_ treatment. Many proteins are closely associated with protein-protein interaction, playing roles in stress response, mitochondria, carbohydrate metabolism, protein synthesis, UPS, RNA metabolism and cytoskeleton, while some proteins are not associated with, acting in GTPase-related proteins, vesicle transport and hydrolases. These findings indicate that many biological processes are regulated by redox pathways. We also studied the kinetics of reversible oxidation, oxidative modifications of redox-sensitive proteins of Prxs, DJ-1, UCH-L3 and Rla0 by proteomic analysis. We also identified the oxidation-induced conformational changes of Prx2 by HDX-MS. These structural changes induce ubiquitination at a newly exposed region to the solvent, and the polyubiquitinated protein, detected as K48-linked polyubiquitin, not K63 linkage, is readily degraded in proteasome and autophagy. We showed that oxidative stress causes conformational changes to occur in the C-terminal region of Prx2, and that K191 residue in this region is the ubiquitination site. These oxidative chemical and structural changes that lead to their degradation, seem to be a new pathway by which homeostasis of redox-sensitive proteins is maintained in the cell, in addition the previously known oxido-reduction cycle of Prxs based on NADPH-thioredoxin reductase-thioredoxin system and reduction of sulfinic acid by sulfiredoxin^35^ (Fig. 8).

A recent review discussed the involvement of H_2_O_2_ and its antioxidant Prxs in various cellular processes^31^. However, the molecular mechanisms by which H_2_O_2_ is sensed and how this action is transduced in cells, are not well understood. In this study, employing 2D-PAGE to separate proteins differentially appearing in cells exposed to mild oxidative stress (0.5 mM H_2_O_2_), we tried to define the target proteins of oxidative stress. Since the cellular responses of H_2_O_2_ stress vary depending on initial concentrations of H_2_O_2_, reducing power of cell type and cell density^41^, optimal H_2_O_2_ concentrations that produce proper oxidative response were carefully determined and used. Target proteins were identified by peptide sequencing using nanoUPLC-ESI-q-TOF tandem MS and redox-sensitive proteins were identified with protein-protein interactome analysis tool STRING, variously functioning in: stress response, mitochondria, carbohydrate metabolism, RNA metabolism, cytoskeleton, protein synthesis and degradation and as ‘not connected’ proteins functioning in vesicle transport and GTPase-associated processes (Fig. 1b). We also showed by 2D-PAGE analysis, that redox-sensitive proteins including Prxs, DJ-1 and UCH-L3 appear in several modified populations, each population responding differently to oxidative stress. Clarification of the relationship between the nature of protein population and their biological function will provide further understanding of the biochemical changes that occur in cells under oxidative stress. We focused on the chemical and structural changes in redox-sensitive proteins (Prxs, DJ-1, UCH-L3, Rla0) in response to H_2_O_2_ treatment. In response to oxidative stress, Prx1 is oxidized and shifts to acidic regions on 2D-PAGE and western analysis^42^, and is newly generated as reduced and basic moiety during recovery by reduction^43^. However, these molecular changes remain to be clarified in terms of the degree of oxidation, because a previous study showed that Prx is regulated by catalytic cycle and that oxidized Prx is reduced again, but not degraded^20^.

This study is the first to demonstrate that redox-sensitive proteins are oxidized to various forms, as identified by combined nanoUPLC-ESI-q-TOF tandem MS and SEMSA, a sensitive approach for detection of low abundant PTMs, and unknown modifications were searched using MOD^i^ and MODmap algorithm^25^,^32^,^33^. Diversely oxidized populations were identified at specific sites, including conversion of Cys to Ser, Dha, sulfonic acid and thiosulfonic acid as described previously^22^. Redox control of degradation of ER proteins seems to occur by formation of disulfide bonds, by mechanisms not well understood. In order to understand how oxidation causes protein conformational changes, we compared the structural changes of control and oxidized Prx2, employing HDX-MS, combined with protein modeling^40^. We found that C-terminal peptide of Prx2 is significantly exposed to solvent by oxidation, which is similar to what happens with oxidized Prx3 as shown in a previous study^44^. Employing mutants, we further investigated whether Lys residues in C-terminal region of Prx2, exposed to protein surface by oxidative stress, are involved in ubiquitination and degradation, and found that Lys191 is readily ubiquitinated with K48-linkage under the oxidative condition (Fig. 7f) and that this ubiquitination induces the degradation of Prx2 in both proteasome and autophagy (Fig. 5).

Protein quality control is achieved by a process that includes new protein synthesis, and folding and refolding, and degradation of proteins damaged by various stresses. Two main substrate dependent proteolytic pathways are known to operate, one in proteasome and the other in autophagy^45^. A previous study showed that oxidized proteins are degraded by ubiquitin-proteasome system (UPS)^46^. However, our present study demonstrates that oxidized Prxs and DJ-1 are degraded in both proteasome and autophagy. Lys48-linked polyubiquitinated Prx2 under oxidative condition can be detected only using both proteasome and autophagy inhibitors. Our results agree with a recent report that there are compensatory mechanisms between autophagy and proteasome degradation and that oxidative stress can induce ROS to facilitate autophagy^47^. Although Lys63 polyubiquitination is suggested as a minor modulator of the oxidative stress response^48^, this study shows that Lys48-linked polyubiquitination is a major modulator of Prx.

There are also two known mechanisms in proteasomal degradation. One, the ubiquitin-26S proteasomal degradation pathway, is considered to be the primary mechanism. The second, the core 20S proteasomal degradation pathway, is now being well-defined. Degradation in the 20S proteasome is possible without ubiquitination, depending on oxidative structural disorder, mutation, or aging^49^,^50^. Some proteins including p53 and p73 are regulated by both ubiquitin-dependent and –independent pathways^51^. In this study, hyperoxidized Prx1 and 2 are ubiquitinated as detected by MS, and then degraded in proteasome and autophagy. Since Prxs are key molecules that maintain redox homeostasis, their amounts are tightly controlled by thioredoxin- and sufiredoxin-dependent reduction, by degradation of hyperoxidized Prxs, and by new synthesis of active protein. Further studies are needed to determine whether oxidized Prxs are also degraded via ubiquitin-independent 20S proteasome machinery.

In the catalytic cycle of 2-Cys Prxs, Prx is oxidized to disulfide by cellular H_2_O_2_ and the disulfide of oxidized Prx is reduced by NADPH-thioredoxin reductase-thioredoxin system. In higher degrees of oxidative stress, hyperoxidized redox-sensitive proteins including Prxs and DJ-1 cannot be reduced by thioredoxin system, but are degraded, and active reduced proteins are newly synthesized during recovery. Although each redox-sensitive protein has different sensitivity to oxidative stress and recovery kinetics^28^, a common pathway maintains homeostasis. Further studies examining the redox sensitivity of each protein based on the structural changes and the degree of oxidation, are needed for understanding the complex interplay between protein oxidation and protein lifespan regulated by protein degradation.

In summary, we showed that redox-sensitive proteins under oxidative stress are oxidized in various ways in dose- and recovery time-dependent manners, and that oxidation of redox-sensitive proteins promotes their degradation in proteasome and autophagy. Employing Prx2 as a model system, we also demonstrated that oxidized Prx2 is readily ubiquitinated at Lys191 residue, which is exposed to solvent by oxidation-induced conformational change, and degraded in proteasome and autophagy. This study suggests a novel molecular regulation pathway for redox-sensitive proteins under oxidative stress. Further studies are required to understand the complex interplay between protein oxidation and protein lifespan, regulated by protein degradation, and to validate the general applicability of this pathway to other redox-sensitive proteins.

## Methods

### Materials

EMEM, penicillin, streptomycin, fetal bovine serum and trypsin were purchased from GIBCO Life Technologies Inc. (Grand Island, NY, USA). Transfection reagent, Effectene was from Qiagen (Hilden, Germany) and LT-1 from Mirus (WI, USA). Monoclonal anti-tubulin antibody and polyclonal anti-DJ-1 antibody were purchased from Santa Cruz Biotechnology (Santa Cruz, CA, USA) and Protein G sepharose beads from GE Healthcare (Giles, United Kingdom). Polyclonal anti-Prx antibodies and anti-Prx-SO_2/3_H antibody were obtained from Ab Frontier (Seoul, Korea). Other biochemicals including monoclonal anti-FLAG antibody (M2), 3-MA (3-methyl adenine), bis-acrylamide, TEMED, ammonium persulfate, sodium dodecyl sulfate (SDS), glycerol, glycine, 2-mercaptoethanol, cycloheximide, deuterium oxide (D_2_O, ≥ 99.9 atom %D), trisodium citrate, TCEP (Tris (2-carboxyethyl)phosphine hydrochloride), porcine pepsin, deoxycholic acid, Na_3_VO_4_ (sodium orthovanadate) and disodium succinate were from Sigma Aldrich (St. Louis, MO, USA), lactacystin from A.G. Scientific (San Diago, USA), trifluoroacetic acid (TFA), formic acid (FA) and HPLC grade acetonitrile from Merck (Darmstadt, Germany) and HPLC grade water from J. T. Baker (PA, USA). Tris-HCl was from Duchefa (Haarlem, the Netherlands) and NP-40 from Amnesco (Ohio, USA).

### Cell culture

Human cervical carcinoma Hela cells and human breast cancer MDA-MB-231 cells from ATCC (VA, USA) were cultured in Eagle’s minimum essential medium (EMEM) supplemented with 10% fetal bovine serum (FBS), 100 μg/mL streptomycin and 100 units/mL penicillin G at 37 °C in an atmosphere of 5% CO_2_ − 95% air.

### Two-dimensional gel electrophoresis

For IEF (isoelectricfocusing), 100 μg of each protein sample were loaded onto the strip gels, rehydrated for 12 h (18 cm, pH 4–7) with rehydration buffer (7 M urea, 2 M thiourea, 2% v/v CHAPS, 2% IPG buffer (pH 4–7). The protein samples were then electrofocused in a manifold cup-loading system with IPGphor (GE Healthcare, Piscataway, NJ, USA), and 2^nd^ dimension was carried out at 15 mA overnight using a PROTEAN II xl 2-D Cell apparatus (BIO-RAD, Hercules, CA, USA) following our previous described procedure^52^.

### Detection of protein spots and image analysis

Each set of gels was silver-stained simultaneously in the same tray. The stained gels were then scanned using an Image Scanner III (GE Healthcare). Spot detection, matching, normalization and quantification were carried out using the Progenesis SameSpots Ver 5.0 (Nonlinear Dynamics) software. For high reliability, same parameters, based on the stringent criteria (fold difference in protein abundance >1.5, *p* value <0.05) were applied to each set of analytical gels. The protein spots showing at least 1.5 fold differences in three replicates were subjected to MS/MS analysis for identifying proteins and their modifications.

### Identification of proteins and post-translational modifications (PTMs), employing nanoUPLC-ESI-q-TOF tandem MS

Peptide sequencings were performed by nanoAcquity™ UPLC™/ESI/MS (SYNAPT™ G2-Si™, Waters Co. UK). The gel spots on 2D-PAGE were destained and digested with trypsin and the resulting peptides extracted as previously described^27^. The peptide extracts were evaporated to dryness in SpeedVac and dissolved in 10% acetonitril solution containing 1.0% formic acid. The dissolved samples were desalted on line prior to separation using trap column (5 μm particle size, NanoEase^TM^ dC_18_, Waters Co., Milford, MA, USA) cartridge. Peptides were separated by chromatography using a C18 reversed-phase 75 μm i.d. × 200 mm analytical column (1.7 μm particle size, BEH130 C18, Waters) with an integrated electrospray ionization PicoTip™ (±10 μm, New Objective, USA). Peptide mixtures (5 μL) were dissolved in buffer A (Water/formic acid; 100:0.1, v/v), injected on a column and eluted by a linear gradient of 5–60% buffer B (ACN/formic acid; 100:0.1, v/v) over 120 min. Initially, the flow rate was set to 250 nL/min and the capillary voltage (2.5 keV) was applied to the nanoUPLC™ mobile phase before spray. Mass analysis was performed on line to SYNAPT™ G2-Si™. The mass spectrometer was programmed to record scan cycles composed of one MS scan followed by MS/MS scans of the 3 ~ 4 most abundant ions in each MS scan. MS parameters for efficient data-dependent acquisition were intensity (>10), number of components (3 ~ 4) to be switched from MS to MS/MS analysis.

Raw data obtained from the mass spectrometer were converted to .pkl files using ProteinLynx Global Server^TM^ (PLGS) 2.3 data processing software (Waters Co., Milford, MA, USA). MS/MS spectra were matched against amino acid sequences in NCBI (USA) and SwissProt using the database search program Mascot (global search engine), ProteinLynx Global SERVER (PLGS) 2.3 (Waters Co., UK).

In order to raise the MS coverage for PTM analysis, SEMSA methodology was employed^25^. The first run analysis, the 4 most abundant precursors were selected for MS/MS analysis. Following positive identification, all identified peptides from database search (Mascot) were non-redundantly excluded in the next run analysis until almost full sequence coverage was obtained. Large numbers and types of potential PTMs were considered. All reported assignments were verified by automatic and manual interpretation of spectra using the database search program Mascot (global search engine), ProteinLynx Global SERVER (PLGS) 2.3 (Waters Co., UK) and MOD^i^ (Korea, http://prix.hanyang.ac.kr/modi/) in a blind mode^32^,^33^.

### Protein degradation assay

MDA-MB-231 cells (3.5 × 10^5^) were exposed to 0.5 mM H_2_O_2_ in HBSS for 1 h, washed and incubated in EMEM supplemented with 10% FBS and the protein synthesis inhibitor, CHX (25 μg/mL) for various recovery times. Degradation rates of Hela cells (1.5 × 10^5^) overexpressing Prx2 wild type and K191R/K196R mutants, and HA-ubiquitin were measured same way as with MDA-MB-231 cells except adding 1 mM sodium pyruvate in media as an additional source of energy, and having protective effects against hydrogen peroxide.

### Down-regulation of Srx using Srx specific siRNA

Specific siRNAs targeting human Srx, oligonucleotides containing small interfering RNA sequences targeting Srx (5′-GGAGGUGACUACUUCUACU-3′); sense 5′-TTCTCCGAACGTGTCACGT-3′ and antisense 5′-ACGTGACACGTTCGGAGAA-3′ for control siRNA were constructed. Transfections with Srx siRNA were carried out using Lipofectamin^®^ RNAiMAX (Invitrogen, CA, USA) according to the manufacturer’s protocol, after 48 h, degradation assay was performed.

### Hydrogen-Deuterium exchange mass spectrometry (HDX-MS)

Purified native and oxidized recombinant Prx2 (about 1 mg/mL) were diluted 20-fold with D_2_O and maintained at 25 °C for various times. Oxidized Prx2 was prepared by incubating in 1 mM H_2_O_2_ for 1 h. The deuterium labeling reaction was quenched by 5 mM Tris (2-carboxyethyl) phosphine (TCEP), pH 2.3. For protein digestion, porcine pepsin (1 mg/mL) was added to each quenched protein sample and incubated at 0 °C for 3 min before injection. Peptic peptides were desalted and separated as previously described^27^. The auto-sampler chamber was set at 5 °C. The trap, analytical column and all tubing were immersed in an ice bath to minimize deuterium back-exchange. Both mobile phase bottles containing 0.1% formic acid were placed on ice. Gradient chromatography was performed at a flow rate 0.6 mL/min and was sprayed on line to nanoAcquityTM/ESI/MS (SYNAPT^TM^ HDMS^TM^, Waters). All mass spectral measurements were taken at: capillary voltage 2.5 keV, cone voltage 35 eV, extraction cone voltage 4.0 eV, and source temperature 80 °C. TOF mode scan was performed in the range of m/z 300–1500 with scan time of 1 s. Peptic peptides were identified with MS/MS analysis. The extent of deuterium incorporation was calculated by monitoring the increase in mass of the isotope distribution for each identified peptide. The theoretical maximum deuterium incorporation value was calculated for each peptide based on the number of exchangeable amides.

### Modeling study of dimeric oxidized form of Prx2

MODELLER 9.9^40^ was used to build an automated homology model of dimeric oxidized form of Prx2. Prx1 of Rattus norvegicus (RnPrx1) which shares sequence identity of 78% with human Prx2, was selected as the template by the BLAST program (http://blast.ncbi.nlm.nih.gov). The three-dimensional structure coordinates of dimeric oxidized form of RnPrx1 (PDB ID: 1QQ2) were obtained from the Brookhaven Protein Data Bank. The input files for MODELLER were the PDB file of the template, the alignment file of the template and target sequences, and Python command script files. Energy minimized models were generated with CHARMM energy parameters. The most reasonable model was selected on the basis of evaluation and the structural quality of the model was verified with PROCHECK^53^.

### Prediction of ubiquitination sites of Prx2

In silico prediction of the ubiquitination sites was performed using UbPred^39^ and two lysine residues (K191, K196) were identified as ubiquitination sites.

### Immunoprecipitation and western blot analysis

Hela cells (7.5 × 10^6^) overexpressing wild type Prx2 and mutants were co-transfected with HA-ubiquitin (HA-Ub) using LT-1. After 24 h, cells were exposed to 0.5 mM H_2_O_2_ in HBSS for 1 h and then recovered in EMEM containing 10% FBS, 2.5 μM lactacystin, 2.5 mM 3-MA and CHX (25 μg/mL) for 12 h. Cells were lysed in RIPA buffer (50 mM Tris, 150 mM NaCl, 0.1% SDS, 0.5% deoxycholate, 1% NP 40, pH 8.0) containing protease inhibitor cocktail (Sigma-Aldrich, USA, aprotinin, PMSF, leupeptin, pepstatin) and phosphatase inhibitor (5 mM Na_3_VO_4_), for 10 min on ice, sonicated for 5 seconds twice, and centrifuged at 12,000 rpm for 15 min. The supernatant was incubated with anti-Flag antibody for 16 h at 4 °C and then with protein G sepharose^TM^ affinity beads (GE Healthcare Bioscience AB, Uppsala, Sweden) for 1 h at 4 °C. The beads were washed six times with 3 mL of RIPA buffer and 3 mL of IP buffer solution (50 mM Tris-Cl, 150 mM NaCl, 0.5% NP-40, pH 7.5) to remove nonspecific binding. The immune complex was solubilized in SDS gel sample buffer, separated by 10% SDS-PAGE, and detected with silver staining or western blot analysis. Chemiluminescence signal was captured using LAS3000 system (Fujifilm, Japan), and each band was quantified using Multi Gauge V3.0 software (Fujifilm, Japan). The sources of antibodies used in this study were: anti-Flag (Sigma, USA), anti-Ub (Millipore, Germany) and anti-tubulin antibodies (Santa cruz, USA).

### Statistical analysis

All data were presented as mean ± SE. Statistical analysis was executed using Origin 8.5 (Origin lab cooperation, USA). Statistical comparisons were performed using two-tailed Student’s t-test between two groups and One-way ANOVA followed by Bonferroni’s post hoc analysis for multiple comparisons among over three groups. P values of under 0.05 were considered statistically significant.

## Additional Information

**How to cite this article**: Song, I.-K. *et al*. Degradation of Redox-Sensitive Proteins including Peroxiredoxins and DJ-1 is Promoted by Oxidation-induced Conformational Changes and Ubiquitination. *Sci. Rep.* **6**, 34432; doi: 10.1038/srep34432 (2016).

## Supplementary Material

Supplementary Information
