# Supplementary material for: Degradation of Redox-Sensitive Proteins including Peroxiredoxins and DJ-1 is Promoted by Oxidation-induced Conformational Changes and Ubiquitination
Source: Sci Rep. 2016 Oct 5;6:34432. doi: 10.1038/srep34432 (PMC5050490; doi:10.1038/srep34432)
Supplement: Supplementary Information [file srep34432-s1.pdf]

Supporting Information

Degradation of Redox Sensitive Proteins including Peroxiredoxins and DJ-1 is Promoted by Oxidation-induced Conformational Changes and Ubiquitination

In-Kang Song[1], Jae-Jin Lee[1], Jin-Hwan Cho[1], Jihye Jeong, Dong-Hae Shin[1], Kong-Joo Lee[1*]

[1] Graduate School of Pharmaceutical Sciences and College of Pharmacy, Ewha Womans University, Seoul 120-750, Republic of Korea

Supplementary Table S1. Identified proteins of H_2O_2-responsive MDA-MB 231 cells afte r 30min of 0.5mM H_2O_2 treatment

Spot No.	SwissProt Accession No.	Matched to species	Identified protein	Mass	pI	Mascot score	Coverage	fold change
Proteins that function in stress response								
2	O60568	PLOD3_HUMAN	Procollagen-lysine,2-oxoglutarate 5-dioxygenase 3	84,731	5.69	254	32	0.211
17	Q9Y265	RUVB1_HUMAN	RuvB-like 1	50,196	6.02	121	16	0.333
19	P14868	SYDC_HUMAN	Aspartyl-tRNA synthetase, cytoplasmic	57,100	6.11	268	40	0.677
22	Q10713	MPPA_HUMAN	Mitochondrial-processing peptidase alpha subunit, mitochondrial precursor	58,216	6.45	45	8	0.906
67	P07900	HS90A_HUMAN	Heat shock protein HSP 90-alpha	84,607	4.94	201	3	1.294
68	P07900	HS90A_HUMAN	Heat shock protein HSP 90-alpha	84,607	4.94	467	13	3.986
70	P02768	ALBU_HUMAN	Serum albumin precursor	69,321	5.92	44	4	1.73
73	P10809	CH60_HUMAN	60 kDa heat shock protein, mitochondrial	61,016	5.70	43	21	1.435
74	P10809	CH60_HUMAN	60 kDa heat shock protein, mitochondrial precursor	61,016	5.70	183	31	1.595
88	Q969H8	CS010_HUMAN	Uncharacterized protein C19orf10 precursor	18,783	6.20	73	8	6.255
M1	P32119	PRDX2_HUMAN	Peroxiredoxin-2	21,878	5.66	104	39.4	0.079
M2	P32119	PRDX2_HUMAN	Peroxiredoxin-2	21,878	5.66	227	45.5	51.96
M3	P30048	PRDX3_HUMAN	Thioredoxin-dependent peroxide reductase, mitochondrial	27,675	7.68	66	14.8	0.041
M4	P30048	PRDX3_HUMAN	Thioredoxin-dependent peroxide reductase, mitochondrial	27,675	7.68	121	41	0.021
M5	P30048	PRDX3_HUMAN	Thioredoxin-dependent peroxide reductase, mitochondrial	27,675	7.68	321	43	52.2
M6	Q13162	PRDX4_HUMAN	Peroxiredoxin-4	30,521	5.86	159	37.3	0.96
M7	Q13162	PRDX4_HUMAN	Peroxiredoxin-4	30,521	5.86	206	62.4	4.723
M8	P30041	PRDX6_HUMAN	Peroxiredoxin-6	25,019	6.00	548	69.7	0.066
M9	P30041	PRDX6_HUMAN	Peroxiredoxin-6	25,019	6.00	431	62.9	8.154
M10	Q99497	PARK7_HUMAN	Protein DJ-1	19,878	6.33	131	79.4	0.457
M11	P30048	PARK7_HUMAN	Protein DJ-1	19,878	6.33	102	36.5	4.403
Proteins that function in mitochondria								
5	P28331	NDUS1_HUMAN	NADH-ubiquinone oxidoreductase 75 kDa subunit, mitochondrial precursor	79,417	5.89	117	16	0.482
9	P31040	SDHA_HUMAN	Succinate dehydrogenase [ubiquinone] flavoprotein subunit, mitochondrial precursor	72,645	7.06	419	23	0.864
21	Q12849	GRSF1_HUMAN	G-rich sequence factor 1	53,093	5.83	125	15	0.587
25	P36957	ODO2_HUMAN	Dihydrolipoyllysine-residue succinyltransferase component of 2-oxoglutarate dehydrogenase complex, mitochondrial precursor	48,609	9.10	83	7	0.265
44	P50213	IDH3A_HUMAN	Isocitrate dehydrogenase [NAD] subunit alpha, mitochondrial precursor	39,566	6.46	64	3	0.875
49	P50213	IDH3A_HUMAN	Isocitrate dehydrogenase [NAD] subunit alpha, mitochondrial precursor	39,566	6.46	53	5	0.205
77	P06576	ATPB_HUMAN	ATP synthase subunit beta, mitochondrial	56,525	5.26	452	70	2.413
84	Q9BWD1	THIC_HUMAN	Acetyl-CoA acetyltransferase, cytosolic	41,324	6.47	61	32	24.23
85	P11177	ODPB_HUMAN	Pyruvate dehydrogenase E1 component subunit beta, mitochondrial	39,208	6.20	426	28	0.309
Proteins that act in carbohydrate metabolism								
8	Q96G03	PGM2_HUMAN	Phosphoglucomutase-2	68,240	6.27	74	10	0.988
13	P36871	PGM1_HUMAN	Phosphoglucomutase-1	61,411	6.30	146	23	0.57
16	P30837	AL1B1_HUMAN	Aldehyde dehydrogenase X, mitochondrial	57,202	6.36	139	31	0.619
48	P09104	ENOG_HUMAN	Gamma-enolase	47,239	4.91	103	7	0.543
50	P37837	TALDO_HUMAN	Transaldolase	37,516	6.36	59	10	0.222
54	P37837	TALDO_HUMAN	Transaldolase	37,516	6.36	215	21	0.433
57	P40925	MDHC_HUMAN	Malate dehydrogenase, cytoplasmic	36,403	6.91	46	8	0.785
58	P15121	ALDR_HUMAN	Aldose reductase	35,830	6.52	111	22	0.526
Proteins that function in translation								
34	Q9UQ80	PA2G4_HUMAN	Proliferation-associated protein 2G4	43,759	6.13	151	10	0.904
45	P08865	RSSA_HUMAN	40S ribosomal protein SA	32,833	4.79	109	14	0.832
52	P05388	RLA0_HUMAN	60S acidic ribosomal protein P0	34,252	5.70	195	38	0.234
53	P05388	RLA0_HUMAN	60S acidic ribosomal protein P0	34,252	5.70	168	40	0.233
M14	P05388	RLA0_HUMAN	60S acidic ribosomal protein P0	34,252	5.72	192	44	1.557
M15	P05388	RLA0_HUMAN	60S acidic ribosomal protein P0	34,252	5.72	222	48	0.207
64	Q9UBQ5	EIF3K_HUMAN	Eukaryotic translation initiation factor 3 subunit 12	25,043	4.81	62	11	0.786
Proteins that function in UPS (ubiquitin proteasome system)								
1	P22314	UBA1_HUMAN	Ubiquitin-like modifier-activating enzyme 1	117,774	5.49	76	6	0.165
69	P46379	BAT3_HUMAN	Large proline-rich protein BAT3	119,334	5.40	74	2	1.564
M12	P15374	UCHL3_HUMAN	Ubiquitin carboxyl-terminal hydrolase isozyme L3	26,166	4.84	277	22	0.372
M13	P15374	UCHL3_HUMAN	Ubiquitin carboxyl-terminal hydrolase isozyme L3	26,166	4.84	81	29	2.986
Proteins that function in RNA metabolism								
6	P61978	HNRPK_HUMAN	Heterogeneous nuclear ribonucleoprotein K	50,944	5.39	83	4	0.824
72	P61978	HNRPK_HUMAN	Heterogeneous nuclear ribonucleoprotein K	50,944	5.39	75	11	1.388
35	P31943	HNRH1_HUMAN	Heterogeneous nuclear ribonucleoprotein H	49,198	5.89	218	35	0.76
76	P31943	HNRH1_HUMAN	Heterogeneous nuclear ribonucleoprotein H	49,198	5.89	58	13	1.265
55	P31942	HNRH3_HUMAN	Heterogeneous nuclear ribonucleoprotein H3	36,903	6.37	50	8	0.566
Proteins that function in vesicle transport								
31	O60664	M6PBP_HUMAN	Mannose-6-phosphate receptor-binding protein 1 (Perilipin-3)	47,018	5.30	185	14	0.636
32	Q99536	VAT1_HUMAN	Synaptic vesicle membrane protein VAT-1 homolog	41,893	5.88	130	21	1.024
71	Q15833	STXB2_HUMAN	Syntaxin-binding protein 2	66,396	6.11	57	8	1.866

Spot No.	SwissProt Accession No.	Matched to species	Identified protein	Mass	pI	Mascot score	Coverage	fold change
Proteins that function in cytoskeleton								
4	P26038	MOES_HUMAN	Moesin	67,778	6.08	128	12	0.509
11	Q16555	DPYL2_HUMAN	Dihydropyrimidinase-related protein 2	62,255	5.95	259	19	0.755
12	Q16555	DPYL2_HUMAN	Dihydropyrimidinase-related protein 2	62,255	5.95	64	17	0.541
20	P60709	ACTB_HUMAN	Actin, cytoplasmic 1	41,710	5.29	94	7	0.814
30	P08670	VIME_HUMAN	Vimentin	53,619	5.05	95	38	0.754
38	P08670	VIME_HUMAN	Vimentin	53,619	5.05	115	17	0.521
42	P08670	VIME_HUMAN	Vimentin	53,619	5.05	192	18	0.551
43	P08670	VIME_HUMAN	Vimentin	53,619	5.05	82	13	0.753
79	P08670	VIME_HUMAN	Vimentin	53,619	5.05	112	18	1.655
80	P08670	VIME_HUMAN	Vimentin	53,619	5.05	159	31	4.466
81	P08670	VIME_HUMAN	Vimentin	53,619	5.05	186	27	5.341
39	P08727	K1C19_HUMAN	Keratin, type I cytoskeletal 19	44,065	5.05	974	67	0.033
40	P08727	K1C19_HUMAN	Keratin, type I cytoskeletal 19	44,065	5.05	2769	78	0.409
75	P35527	K1C9_HUMAN	Keratin, type I cytoskeletal 9	62,092	5.14	2172	43	3.562
56	P68363	TBAK_HUMAN	Tubulin alpha-ubiquitous chain	50,120	4.94	258	29	0.457
86	P19105	MLRM_HUMAN	Myosin regulatory light chain 2, nonsarcomeric	19,781	4.65	140	24	1.466
87	P19105	MLRM_HUMAN	Myosin regulatory light chain 2, nonsarcomeric	19,781	4.65	109	30	1.387
Proteins that are realted to GTPase								
24	Q07960	RHG01_HUMAN	Rho GTPase-activating protein 1	50,404	5.85	70	16	0.591
27	P50395	GDIB_HUMAN	Rab GDP dissociation inhibitor beta	50,631	6.10	179	38	0.896
61	Q8NBT2	SPC24_HUMAN	Kinetochore protein Spc24	22,429	4.65	162	28	0.574
65	P52565	GDIR_HUMAN	Rho GDP-dissociation inhibitor 1	23,193	5.01	360	27	0.517
Proteins that have hydrolase activity								
36	P23526	Q1RMG2_HUMAN	Adenosylhomocysteinase	47,685	5.92	121	13	0.231
66	Q9BY32	ITPA_HUMAN	Inosine triphosphate pyrophosphatase	21,432	5.50	111	25	8.582
83	P35237	SPB6_HUMAN	Serpin B6	42,594	5.18	93	19	1.534
Proteins that function in RNA binding								
3	P81605	DCD_HUMAN	Dermcidin precursor	11,277	6.09	147	25	0.501
7	P09960	LKHA4_HUMAN	Leukotriene A-4 hydrolase	69,241	5.79	58	11	0.824
14	P48444	COPD_HUMAN	Coatomer subunit delta	57,174	5.89	101	21	0.669
15	Q9NUU7	DD19A_HUMAN	ATP-dependent RNA helicase DDX19A	53,941	6.20	76	3	0.737
23	P18031	PTN1_HUMAN	Tyrosine-protein phosphatase non-receptor type 1	49,935	5.88	46	11	0.458
28	P13489	RINI_HUMAN	Ribonuclease inhibitor	49,941	4.71	178	31	0.261
29	P13489	RINI_HUMAN	Ribonuclease inhibitor	49,941	4.71	89	7	0.731
59	P25786	PSA1_HUMAN	Proteasome subunit alpha type-1	29,537	6.15	63	14	0.349
Proteins that function in ion binding								
10	P13674	P4HA1_HUMAN	Prolyl 4-hydroxylase subunit alpha-1	61,011	5.70	407	16	0.846
37	Q15293	RCN1_HUMAN	Reticulocalbin-1 precursor	38,866	4.88	112	13	0.58
62	Q08623	HDD1A_HUMAN	Haloacid dehalogenase-like hydrolase domain-containing protein 1A	23,715	5.15	44	5	0.763
63	P05109	S10A8_HUMAN	Protein S100-A8	10,828	6.50	64	11	0.654
Proteins that act in amino acid biosynthesis								
18	Q9UHG3	PCYOX_HUMAN	Prenylcysteine oxidase precursor	56,604	5.80	116	14	0.868
33	P04181	OAT_HUMAN	Ornithine aminotransferase, mitochondrial precursor	48,504	6.57	142	16	0.232
60	Q9NVS9	PNPO_HUMAN	Pyridoxine-5'-phosphate oxidase	29,969	6.61	64	5	0.379
78	Q9HDC9	APMAP_HUMAN	Adipocyte plasma membrane-associated protein	46,451	5.82	89	18	1.655
Proteins that function in protein complex binding								
46	P40121	CAPG_HUMAN	Macrophage capping protein	38,494	5.82	76	3	0.444
47	P40121	CAPG_HUMAN	Macrophage-capping protein	38,494	5.82	130	11	0.468
51	O43765	SGTA_HUMAN	Small glutamine-rich tetratricopeptide repeat-containing protein A	34,042	4.79	46	8	0.232
Protein that functions in lipid metabolism								
26	P22307	NLTP_HUMAN	Nonspecific lipid-transfer protein	58,956	6.44	42	3	0.869
Protein that has endopeptidase activity								
41	P30740	ILEU_HUMAN	Leukocyte elastase inhibitor	42,715	5.90	252	23	0.192
82	P30740	ILEU_HUMAN	Leukocyte elastase inhibitor	42,715	5.90	144	18	1.17

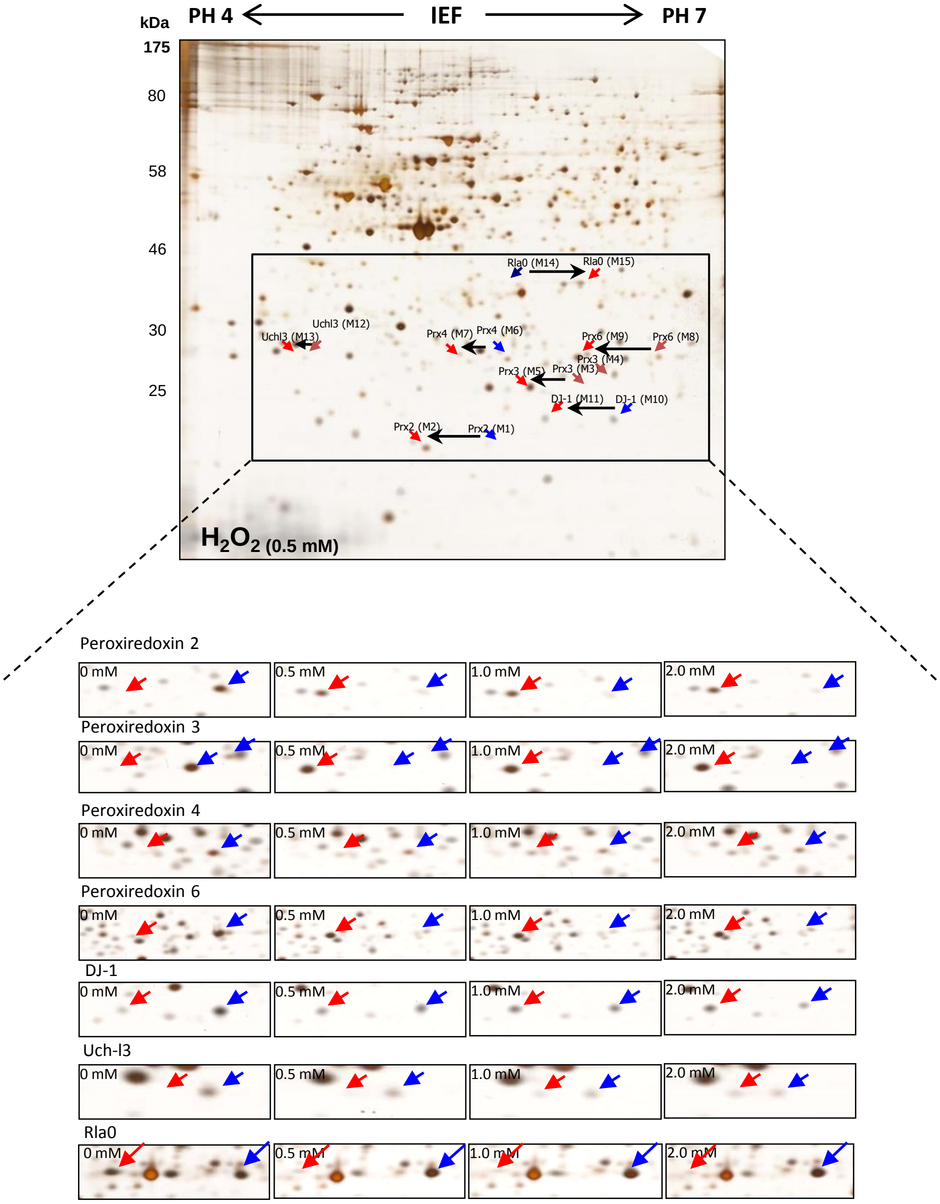

Supplementary Figure S1. 2D-gel images of Prxs, DJ-1, UCH-L3 and Rla0 in MDA-MB-231 cells treated with various concentrations of H_2O_2.

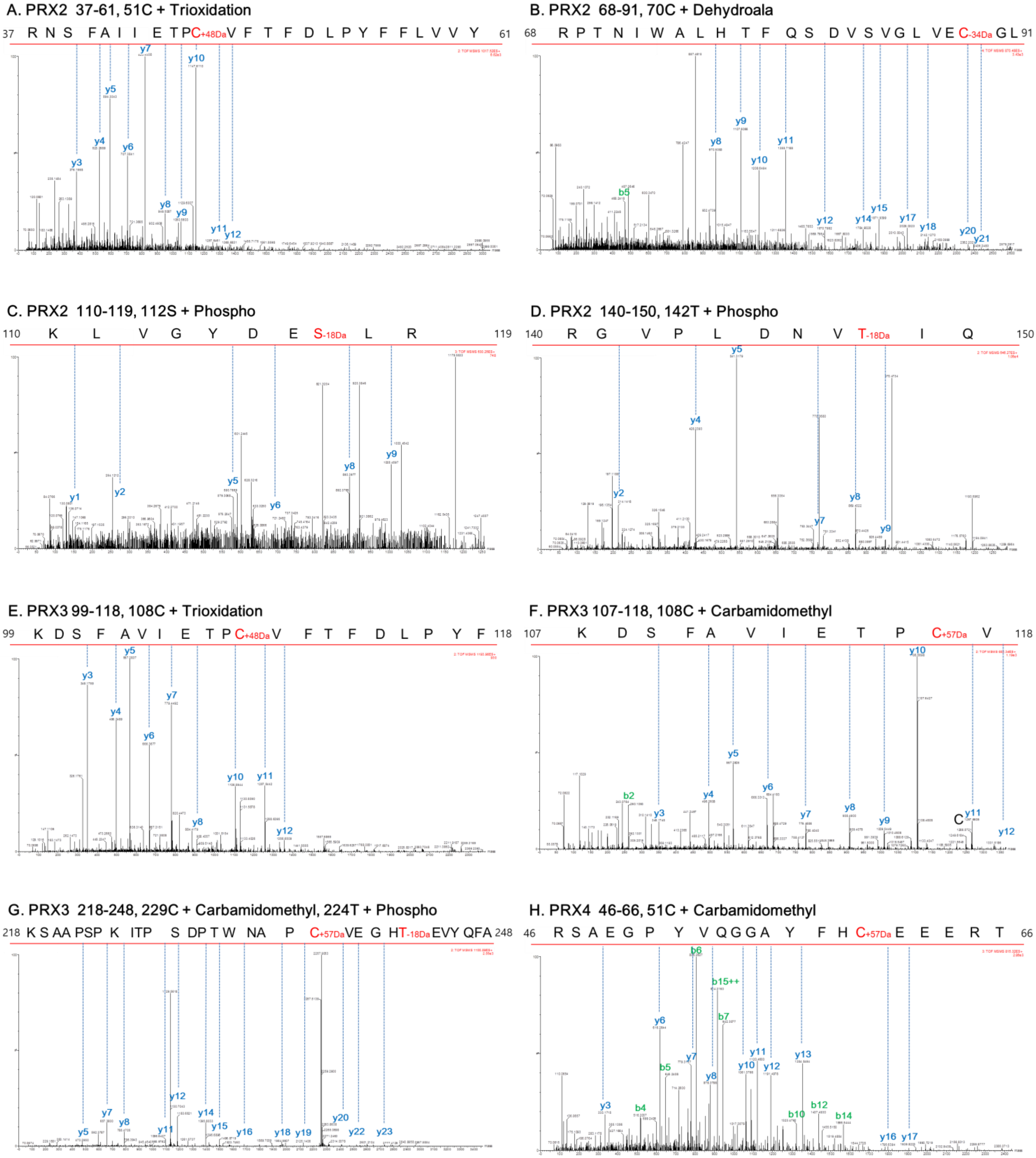

Supplementary Figure S2. Representative MS/MS spectra of identified modifications of Prxs and DJ-1

Song IK *et al*. Supplementary Fig. 2

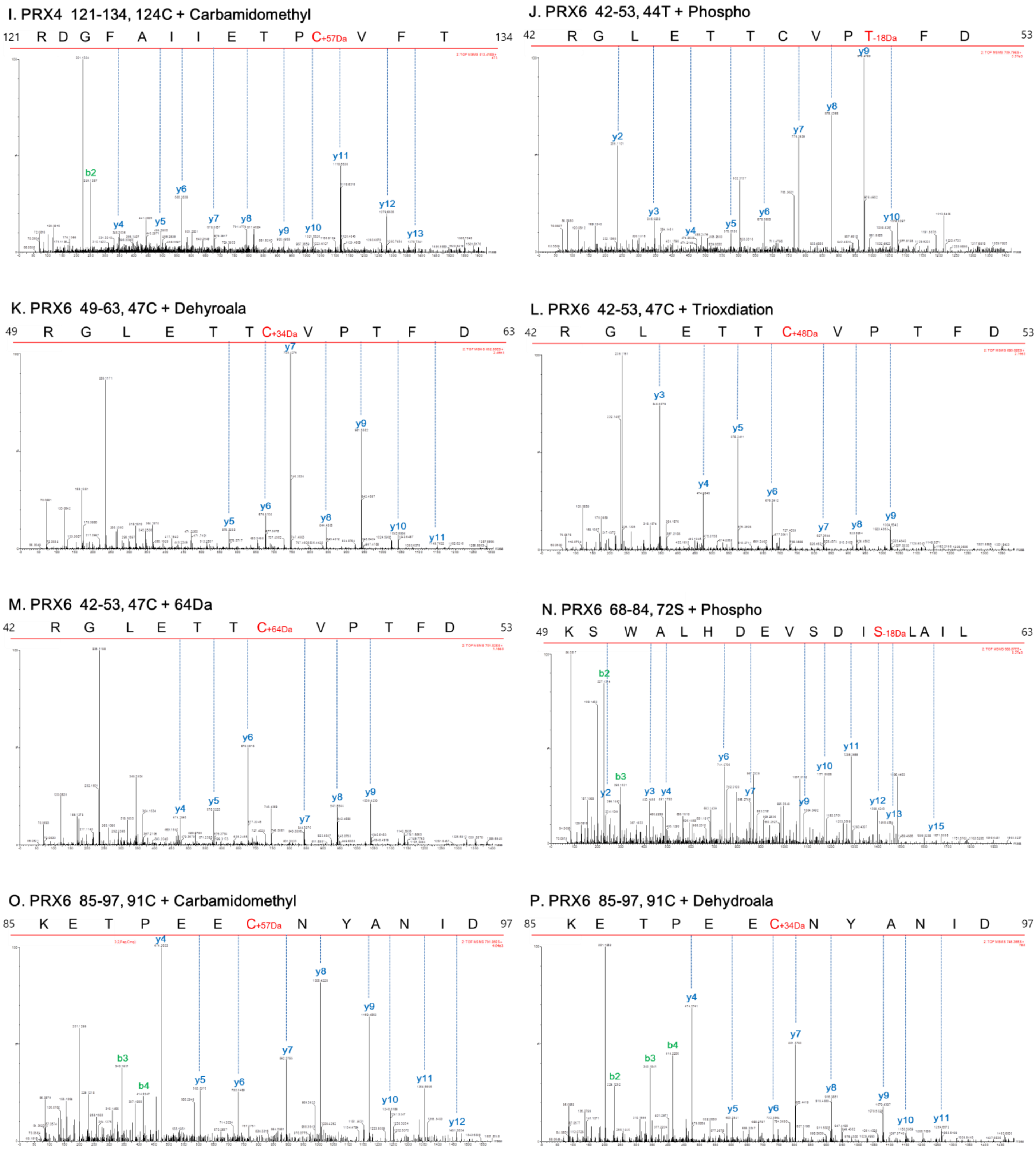

Song IK *et al*. Supplementary Fig. S2

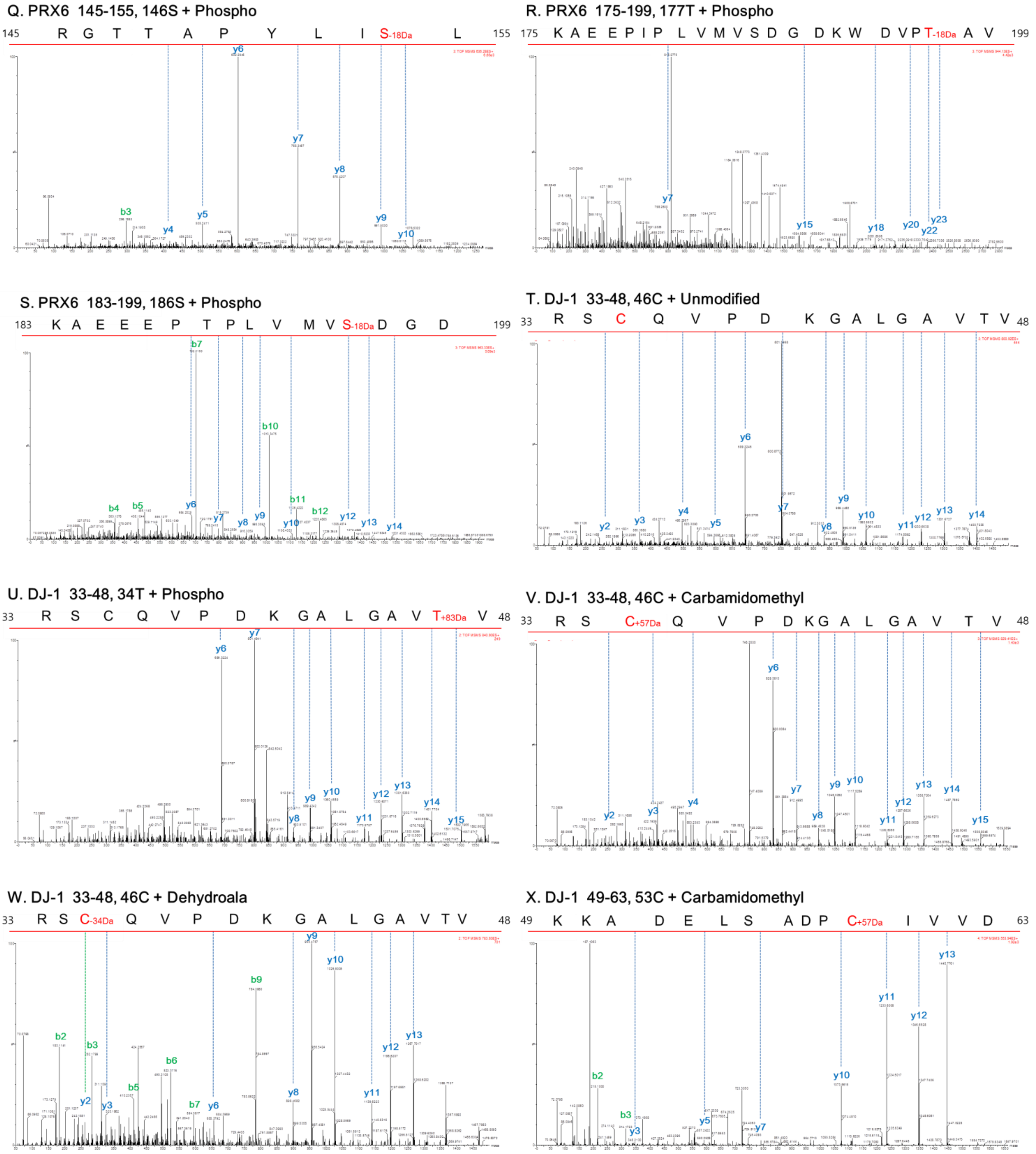

Song IK *et al*. Supplementary Fig. 2

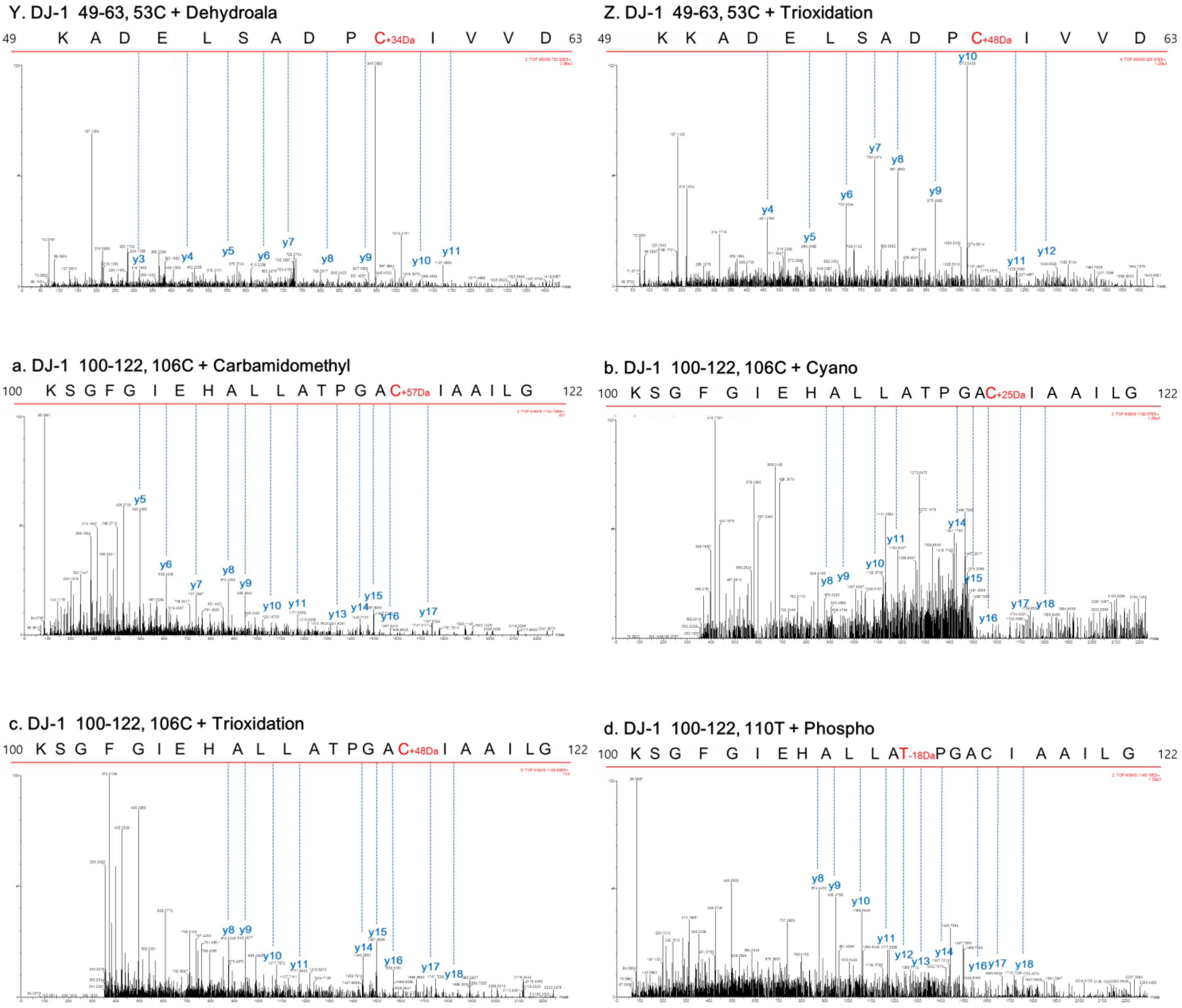

Song IK *et al*. Supplementary Fig. S2

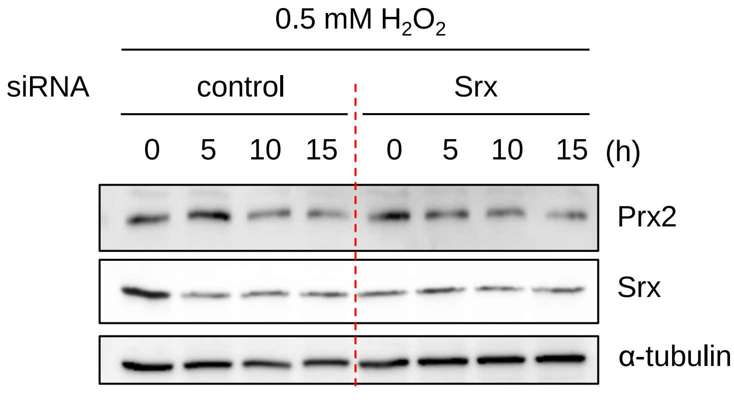

Supplementary Figure 3. Effect of Srx knock-down on degradation of oxidized Prx2 in MDA-MB-231 cells. Forty-eight hours after transfection with a control siRNA or human Srx siRNA (500 nM), MDA-MB-231 cells were incubated with 0.5 mM H_2O_2 and were incubated in EMEM supplemented with 10% FBS, with CHX (25 µg/mL) for indicated time. (Srx antibody was kindly provided by Prof. Chang TS at Ewha womans university.)

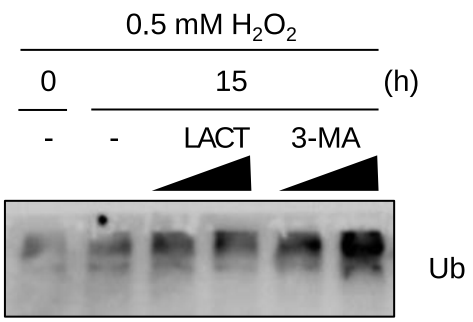

Supplementary Figure S4. Total amount of Ub-proteins were accumulated by proteasome inhibitor lactacystin and autophagy inhibitor 3-MA. MDA-MB-231 cells treated with 0.5 mM H_2O_2 in HBSS for 1 h were recovered in EMEM supplemented with 10% FBS, CHX (25 μg/mL), and lactacystin (1 and 5 mM), an irreversible proteasome inhibitor, or 3-MA (1 and 5 mM), an autophagy inhibitor, for 15 h.

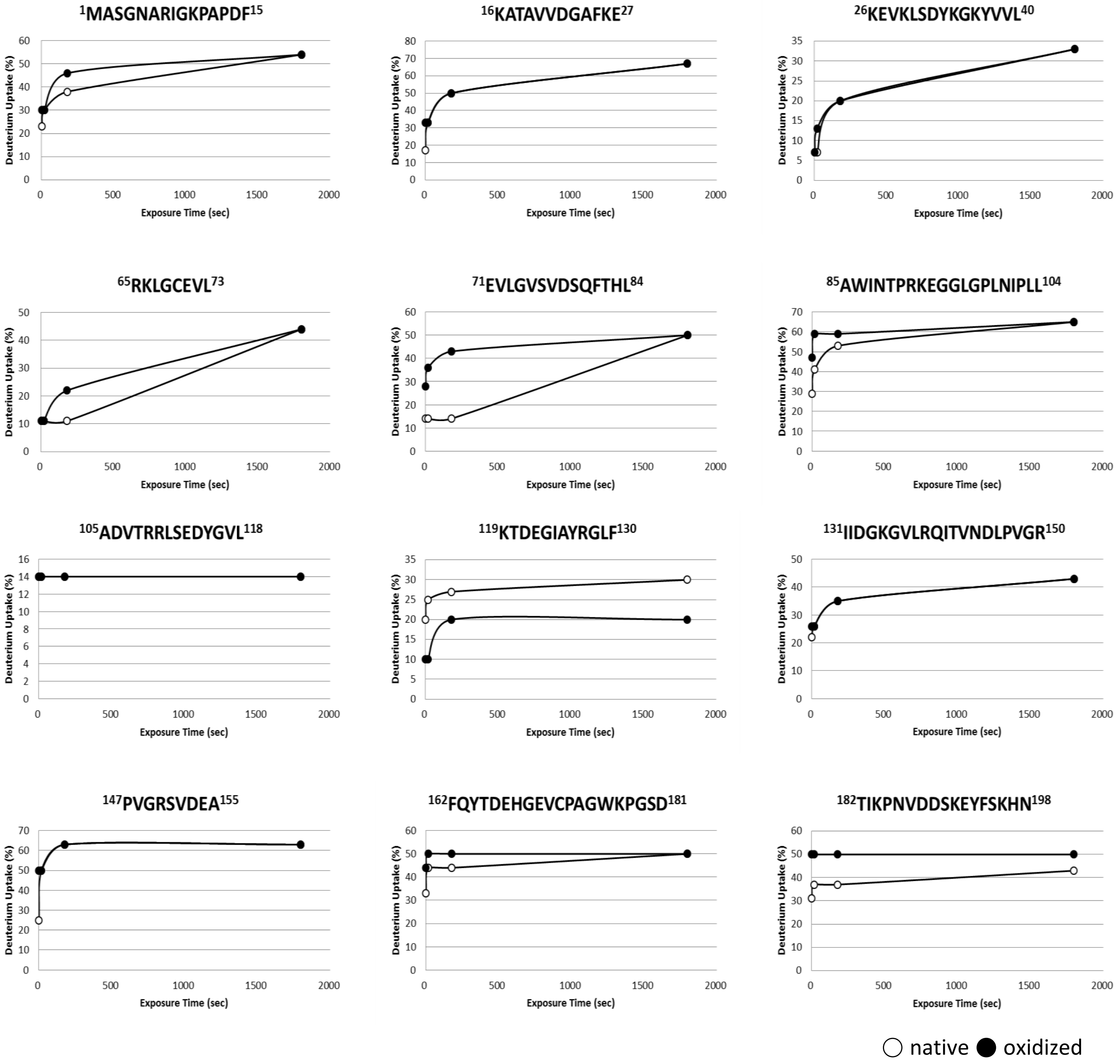

Supplementary Figure S5. Hydrogen/deuterium exchange kinetics of each peptide between native and oxidized Prx2.

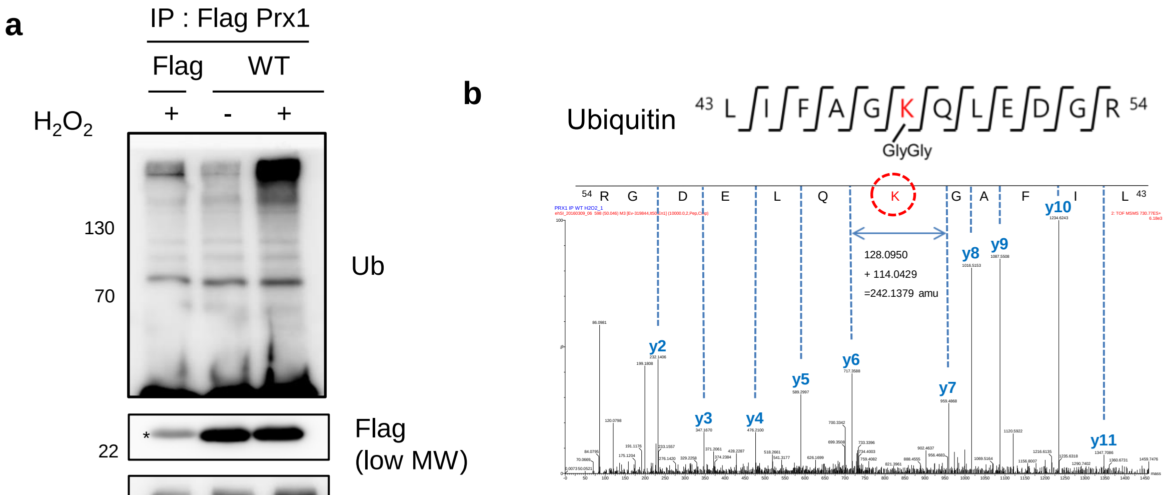

Site/Sequence	m/z	Mr(calc)	Delta	Nomical mass difference (Da)	Position	Modification
43-54 LIFAGKQLEDGR	730.8961(2+)	1459.7776	-0.0007	+114.0429	K48	GlyGly

Supplementary Figure S6. (a) Prx1 is also ubiquitinated as well as Prx2 in response to H_2O_2. Same experiment was performed for Prx1 as that in Figure 5D. *: non-specific band from IgG antibody. (b) Lys-linkage of polyubiquitinated proteins were identified by MS/MS. MS/MS spectra of Lys48-linked ubiquitin peptide is shown.
